# Artificial intelligence in the biomechanics of knee osteoarthritis: biomechanical phenotyping, risk stratification, diagnosis, treatment and clinical translation—a narrative review

**DOI:** 10.3389/fbioe.2026.1937199

**Published:** 2026-09-24

**Authors:** Baihe Zhang, Xinyue Yu, Zipei Zhang, Xiaoling Zhou, Wenhai Zhao, Changwei Zhao

**Affiliations:** 1 Changchun University of Chinese Medicine, Changchun, Jilin, China; 2 The Affiliated Hospital of Changchun University of Chinese Medicine, Changchun, Jilin, China

**Keywords:** artificial intelligence, biomechanical phenotyping, biomechanics, clinical translation, finite element analysis, gait analysis, knee osteoarthritis, wearable sensing

## Abstract

Structural degeneration, pain and functional limitation in knee osteoarthritis (KOA) often diverge, which suggests that radiographic grading alone cannot explain the heterogeneous clinical course of the disease. Lower-limb alignment, gait strategy, muscle function, joint contact forces and real-world activity exposure jointly shape KOA onset, progression and treatment response. Artificial intelligence (AI) methods provide tools for integrating multimodal imaging, gait, wearable-sensor and clinical data, and can be combined with finite element analysis (FEA) and other computational biomechanical models. Conceptually, we propose an evidence-to-action framework that distinguishes AI-supported measurement, longitudinal prediction and intervention or decision utility, while separating AI-native methods from non-AI digital and computational biomechanical technologies. This structured narrative review synthesizes and critically interprets evidence on artificial intelligence in knee osteoarthritis from a biomechanical perspective, with an emphasis on biomechanical phenotyping across imaging, gait, wearable-sensor and clinical data, together with longitudinal risk stratification, diagnosis, progression prediction, rehabilitation feedback, perioperative management and clinical translation. Available studies indicate that artificial intelligence can support cartilage and meniscus segmentation, imaging-based grading, biomechanical load estimation, preoperative planning, and rehabilitation monitoring, although the strength and clinical maturity of the evidence vary substantially across these applications. Multimodal AI models have identified imaging, biomechanical, sensor-derived, and clinical features associated with structural progression, pain, and functional outcomes, but these associations should not be interpreted as causal or clinically actionable without longitudinal, external, and prospective validation. However, limited external validation, heterogeneous data acquisition, label bias, limited causal interpretability, limited use of patient-centered outcomes and poor workflow integration continue to constrain clinical use. Future AI-enabled KOA assessment should be grounded in biomechanical mechanisms and oriented toward patient outcomes and actionable clinical decisions. Translation will require standardized multimodal data, prospective validation and real-world utility evaluation.

## 1 Introduction

Knee osteoarthritis is a major cause of pain and disability in middle-aged and older adults. Its incidence is increasing as populations age (Kloppenburg et al., 2025). The clinical burden arises from the long duration of disease, broad functional impact and escalating treatment needs (GBD 2021 Osteoarthritis Collaborators, 2023). Patients commonly experience knee pain, reduced walking capacity, fear of falling, restricted daily activity and impaired quality of life (Tang et al., 2025). In advanced disease, they may undergo total knee arthroplasty (TKA) or unicompartmental knee arthroplasty (UKA). The Kellgren-Lawrence (KL) grade provides a shared language for epidemiology and clinical communication. However, it is a coarse marker of structural damage and cannot fully capture early tissue change, pain mechanisms, dynamic loading or treatment response (Mahmoudian and Haugen, 2026). Recent biomechanical and mechanistic reviews therefore frame KOA as a complex disease process involving mechanical loading, tissue vulnerability and systemic metabolic inflammation (Diamond et al., 2024; Guan and Mei, 2026).

Early AI studies in KOA mainly focused on classifying radiographs or MRI scans to recognize disease severity or support image interpretation. As automated MRI scoring, cartilage and meniscus segmentation, radiomics, gait recognition, wearable monitoring and biomechanical modeling have developed, the scope of AI has expanded to tissue phenotype quantification, longitudinal progression prediction, identification of treatment candidates and postoperative functional assessment (McDonald et al., 2025). Deep learning now provides a relatively mature methodological spectrum for osteoarthritis imaging, and automated MRI scoring may reduce reliance on some repetitive manual measurements. Model generalizability and clinical interpretability nevertheless remain major bottlenecks (Kijowski et al., 2023; Jarraya et al., 2024).

The field is moving from single-modality recognition toward multimodal integration. Radiographs provide information on joint space, osteophytes and alignment. MRI can reveal changes in cartilage, menisci, subchondral bone and synovium. Three-dimensional gait analysis and inertial measurement units (IMUs) record dynamic loading. Wearable insoles and mobile devices capture load exposure during daily life. FEA and musculoskeletal models can estimate tissue stress and contact force (Mapinduzi et al., 2026). Unlike earlier reviews that focused mainly on imaging classification, severity grading or individual prediction tasks, this review adopts an evidence-to-action framework for AI in KOA. First, can AI stably measure biomechanical phenotypes that are difficult to quantify in routine clinical assessment? Second, can these phenotypes predict KOA onset, progression, pain or functional impairment? Third, can model outputs change screening, follow-up, rehabilitation prescription, treatment allocation, preoperative planning or shared decision-making? These questions reflect increasing proximity to clinical action rather than a simple hierarchy of methodological quality. AI outputs can become interpretable precision-care tools only when the measured object, validation level and clinical action are specified together (Mononen et al., 2024).

We therefore review AI applications in KOA from a biomechanical perspective. This article is a structured narrative review rather than a systematic review or meta-analysis. Its purpose is to map, integrate, and critically interpret heterogeneous evidence across imaging, gait analysis, wearable sensing, computational biomechanics, rehabilitation, perioperative management, and clinical translation. PRISMA-style reporting elements are used to improve transparency of literature identification and selection, but the review should not be interpreted as a fully PRISMA-compliant systematic review. We first outline the biomechanical basis of KOA. We then organize four evidence pathways: imaging, gait, wearable sensing and computational simulation. We further discuss how AI may identify KOA risk phenotypes and candidate modifiable targets, and we assess its roles in diagnosis and prediction, treatment and rehabilitation, perioperative management and clinical translation (Figure 1). The aim is not to catalog AI applications. Rather, it is to develop a translational evidence framework that links measurable biomechanical phenotypes to risk stratification and clinical action, while distinguishing the interpretive boundaries of cross-sectional association, longitudinal prediction, intervention evaluation and real-world implementation.

**FIGURE 1:**
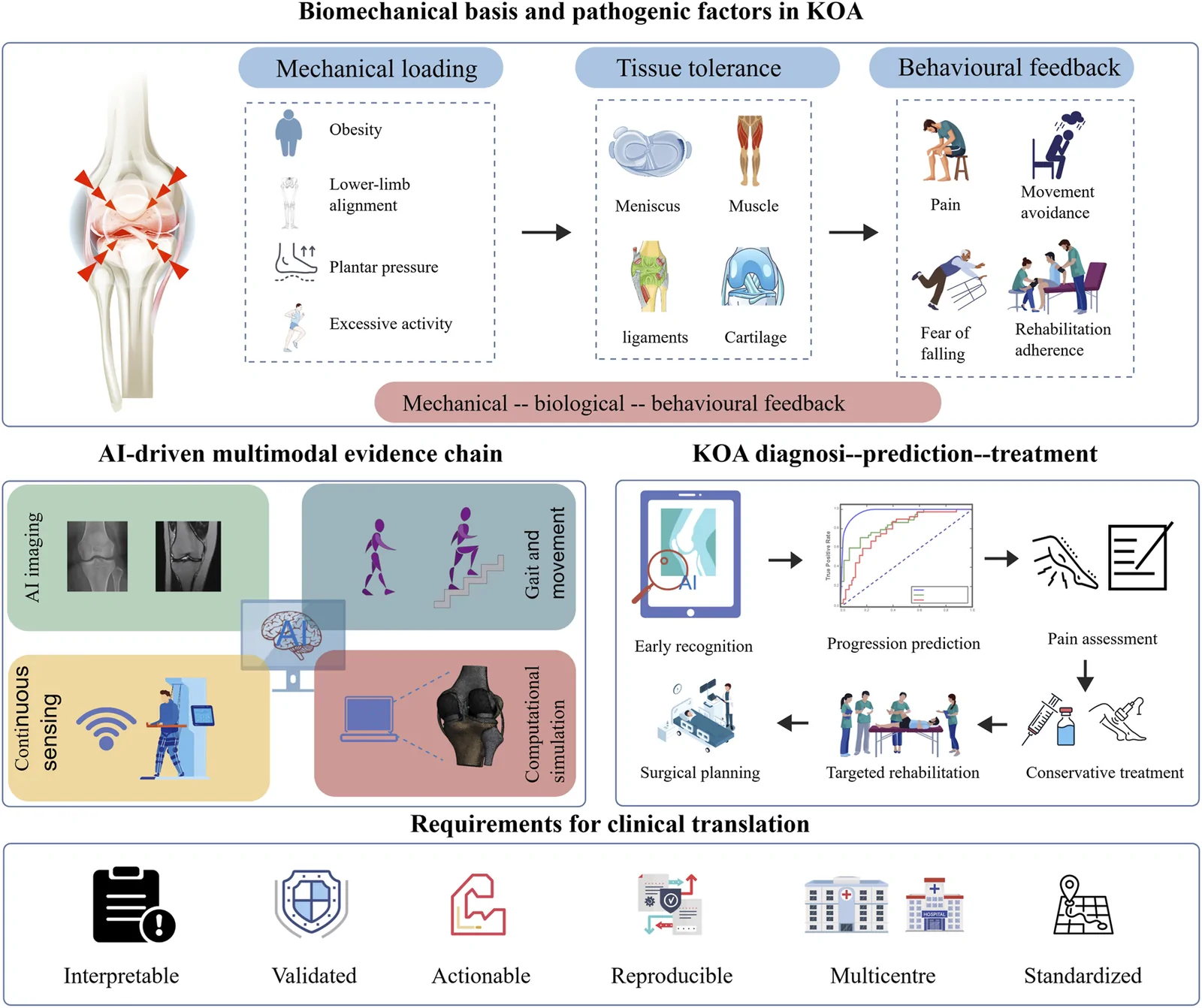
Logic framework of the review. The figure summarizes the pathway from the biomechanical basis of knee osteoarthritis to AI-supported measurement, phenotyping, risk stratification and clinical translation. Multimodal inputs include radiography and magnetic resonance imaging, gait and joint-loading measurements, wearable-sensor data, computational biomechanical models, clinical variables and patient-reported outcomes. AI applications are organized into imaging analysis, gait and wearable sensing, AI-enabled computational modeling, diagnosis and progression prediction, rehabilitation feedback and perioperative decision support. These applications are interpreted according to their evidence level, ranging from technical feasibility and cross-sectional association to longitudinal prediction and intervention or decision utility. Representative studies and their methodological characteristics are summarized in Tables 1–5 and Supplementary Material 4. Conventional finite-element analysis, musculoskeletal modeling, wearable sensing, robotic rehabilitation and internet-based rehabilitation are not considered AI unless AI performs a substantive analytical, predictive or adaptive function.

## 2 Methods

### 2.1 Narrative review design and literature search

This study was designed as a structured narrative review of the interdisciplinary literature at the intersection of artificial intelligence, biomechanics, and the diagnosis and management of knee osteoarthritis. The review was intended to map and critically interpret heterogeneous evidence rather than to answer a single narrowly defined clinical question, estimate a pooled diagnostic or prognostic performance measure, or quantify a treatment effect. Accordingly, the review was not conducted as a systematic review or meta-analysis. Nevertheless, structured database searching, operational eligibility criteria, transparent reporting of record counts, and a PRISMA-style flow diagram were used to improve the transparency and reproducibility of the narrative synthesis.

Systematic database searches used to support this narrative review were conducted in PubMed, Web of Science Core Collection, and the China National Knowledge Infrastructure. The final search was performed on 1 July 2026, and covered records published between 1 January 2010, and 1 July 2026. Search concepts included knee osteoarthritis; artificial intelligence, machine learning, deep learning, neural networks, computer vision, and radiomics; and imaging, gait analysis, wearable sensing, biomechanics, finite element analysis, musculoskeletal modeling, digital twins, diagnosis, prediction, treatment, rehabilitation, and arthroplasty. The complete database-specific search strategies, search interfaces, search dates, and record counts are provided in Supplementary Material 1.

Structured database searches were conducted in PubMed, Web of Science Core Collection, and the China National Knowledge Infrastructure on 1 July 2026, covering records published from 1 January 2010, to 1 July 2026. The searches retrieved 600 records from PubMed, 1,857 records from Web of Science Core Collection, and 461 records from the China National Knowledge Infrastructure, yielding a total of 2,918 records before deduplication. After deduplication, title-and-abstract screening, and full-text relevance assessment, a total of 518 articles were ultimately included in the narrative synthesis.

### 2.2 Study eligibility and selection process

After deduplication, records were assessed sequentially by title and abstract screening and, where potentially eligible, by full-text review. Eligibility was determined according to the population or data source, methodological relevance, outcome relevance, and sufficiency of reporting.

Studies providing core evidence were eligible when they met the following criteria: (1) they included individuals with knee osteoarthritis, individuals at risk of knee osteoarthritis, or knee osteoarthritis imaging, gait, sensor, or clinically relevant biomechanical datasets; (2) they evaluated an artificial-intelligence-native method, including machine learning, deep learning, neural networks, computer vision, or radiomics, or an AI-enabled analytical pipeline; (3) they addressed at least one review domain, including structural or biomechanical phenotype measurement, diagnosis, disease grading, progression prediction, pain or functional outcomes, treatment-response prediction, rehabilitation feedback, perioperative planning, postoperative recovery, or clinical implementation; and (4) they provided sufficient methodological and outcome information to permit interpretation.

Non-AI computational or digital studies, including conventional finite element analysis, musculoskeletal modeling, wearable monitoring, robotic rehabilitation, and internet-based rehabilitation, were eligible only when they provided relevant mechanistic, comparator, or implementation context. Such studies were not treated as direct evidence of AI effectiveness. Reviews, methodological papers, clinical protocols, and regulatory or implementation documents were used for contextual synthesis where relevant but were not interpreted as primary evidence of diagnostic, prognostic, or treatment efficacy.

Studies were excluded when: (1) knee osteoarthritis data could not be separated from other joint disorders or populations; (2) the study was limited to animal, cadaveric, or *in vitro* research without a clinically interpretable knee osteoarthritis application; (3) it described a generic algorithm or technical platform without knee osteoarthritis-specific evaluation; (4) methodological or outcome reporting was insufficient for interpretation; (5) only a conference abstract or similarly incomplete report was available; (6) the report duplicated another publication without providing materially different data or analyses; or (7) a non-AI digital or computational technology did not contribute directly to the biomechanical or translational framework of the review.

Representative studies were selected for detailed narrative discussion and tabulation according to their relevance to the prespecified review domains, methodological contribution, biomechanical interpretability, validation strategy, outcome relevance, and proximity to a potential clinical action. Selection was not based solely on the magnitude of reported model performance.

Dual independent screening was not performed because the review was originally designed as a structured narrative synthesis rather than a systematic review. Consequently, inter-rater agreement was not calculated. This limitation is stated explicitly in the Limitations section. The numbers of records at each stage of the selection process and the main reasons for full-text exclusion are reported in a PRISMA-style flow diagram (Supplementary Material 2).

English- and Chinese-language full-text sources were eligible. Chinese-language publications were retained when they provided relevant information on locally developed artificial-intelligence methods, computational biomechanics, rehabilitation assessment, manual therapy evaluation, or perioperative implementation. Supplementary Material 3 provides brief English annotations of their principal methods and findings. Because several Chinese-language sources are not indexed in major international databases and may require access through the CNKI or institutional repositories, their independent verifiability may be limited. These sources were therefore interpreted cautiously and were not used as the sole basis for strong claims of AI efficacy or clinical utility.

### 2.3 Evidence synthesis and translational interpretation framework

Given the heterogeneity of study designs and clinical questions, evidence was synthesized narratively and organized according to its role in the pathway from measurement to clinical action. For the purpose of translational interpretation, studies were grouped into three broad stages: technical feasibility and cross-sectional association, longitudinal prediction and risk stratification, and intervention or decision utility (Figure 2). These stages represent increasing proximity to clinical action rather than a universal hierarchy of methodological quality.

**FIGURE 2:**
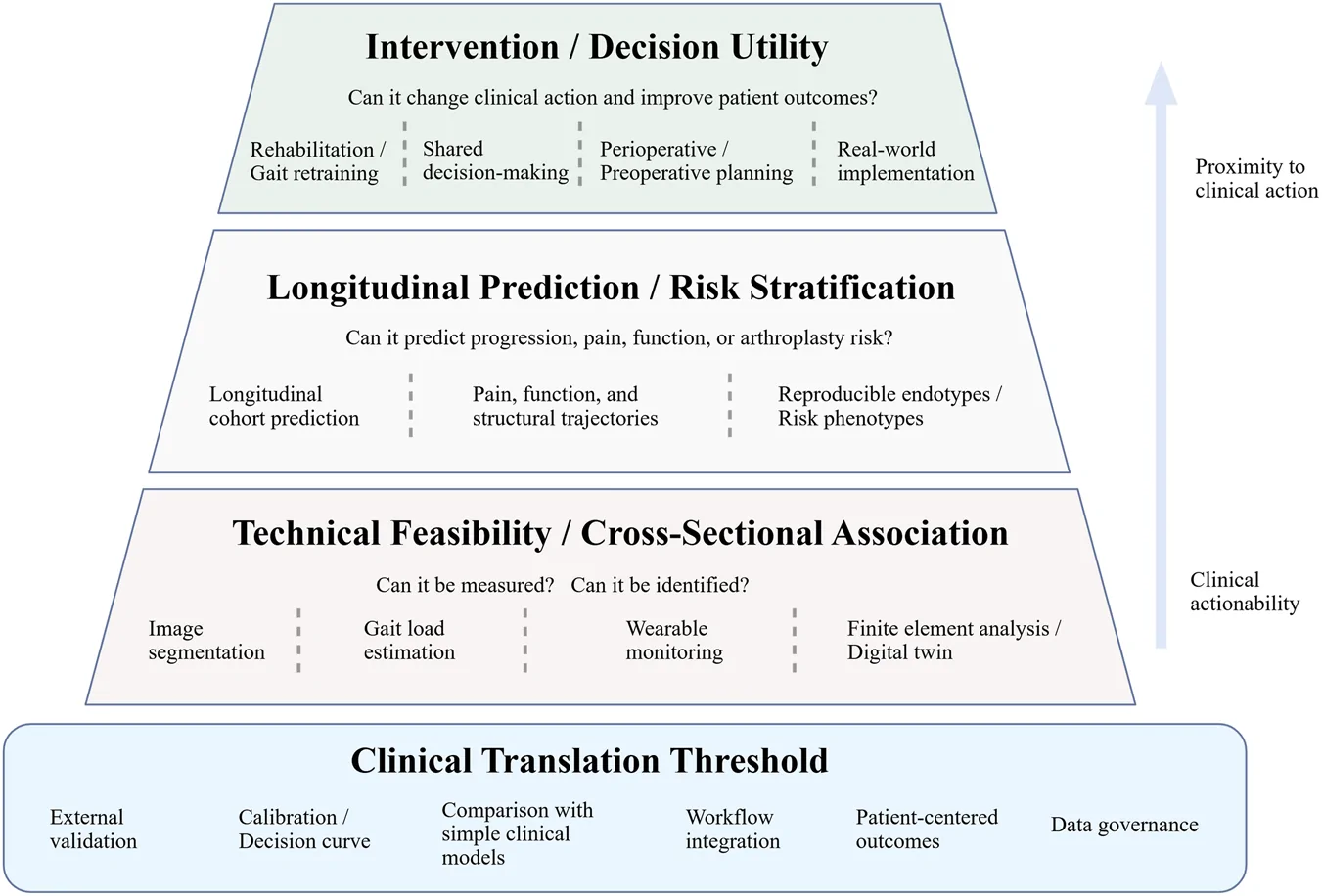
Translational evidence map and interpretive boundaries in AI-enabled knee osteoarthritis biomechanics. The reviewed evidence is organized into three stages according to its proximity to clinical action. The first stage, technical feasibility and cross-sectional association, includes image segmentation, disease classification, biomechanical-variable estimation, sensor deployment and computational simulation. For example, externally validated cartilage-segmentation models demonstrate technical performance but do not by themselves establish improved clinical outcomes (Yao et al., 2026). The second stage, longitudinal prediction and risk stratification, includes models predicting incident knee osteoarthritis, structural progression, pain, functional decline or arthroplasty-related outcomes. External validation of a progression model across the Osteoarthritis Initiative and Multicenter Osteoarthritis Study represents this level of evidence (Lee et al., 2025). The third stage, intervention and decision utility, evaluates whether model outputs influence rehabilitation, treatment selection, shared decision-making or patient-relevant outcomes, as illustrated by AI-supported gait feedback and digital-twin-informed decision support (Wang et al., 2021; Jayakumar et al., 2025). These stages form an evidence-to-action continuum and should not be interpreted as a universal ranking of methodological quality. Validity within each stage also depends on study design, sample size, external validation, calibration and risk of bias.

The first stage included studies of image segmentation, classification, biomechanical-variable estimation, sensor deployment and computational simulation. These studies primarily address whether a phenotype, signal or mechanical quantity can be measured, reconstructed or predicted with acceptable technical performance. Cross-sectional associations identified at this stage were interpreted as risk markers or mechanistic hypotheses rather than evidence of causality.

The second stage included longitudinal studies that predicted structural progression, pain, functional decline, treatment response or arthroplasty-related outcomes. These studies address whether a model can stratify future risk across a defined time horizon. Their value depends not only on discrimination but also on calibration, external validation, robustness across populations and the extent to which model outputs provide information beyond readily available clinical variables.

The third stage included intervention studies, decision-support studies, prospective validation and real-world implementation research. These studies provide more direct evidence of clinical utility by testing whether model outputs can influence treatment selection, rehabilitation, shared decision-making, perioperative planning, workflow or patient-relevant outcomes. However, placement within this stage does not by itself imply higher methodological quality than a well-conducted multicenter diagnostic or prognostic study. Study design, risk of bias, validation quality and sample size must still be considered separately.

To avoid conflating AI with broader digital or computational technologies, we distinguished among AI-native methods, AI-enabled computational pipelines, non-AI computational biomechanics, and digital technologies without an inherent AI component. AI-native methods included machine learning, deep learning, neural networks and radiomics-based prediction models. AI-enabled pipelines included applications in which AI performed a substantive analytical or decision-support function, such as automated segmentation feeding biomechanical modeling, AI-assisted preoperative planning or AI-supported clinical decision-making. Conventional finite element analysis, musculoskeletal modeling, wearable sensing, robotic rehabilitation and internet-based rehabilitation were not considered AI by default. Such non-AI studies were included only when they provided mechanistic, comparator or implementation context and were not interpreted as evidence of AI efficacy.

Within this framework, cross-sectional studies were interpreted primarily as phenotype-identification or hypothesis-generating evidence; longitudinal studies as prediction or stratification evidence; and intervention or implementation studies as evidence more directly relevant to clinical utility. This framework also guided the interpretation of representative studies in the tables, with particular attention to the distinction between technical performance, predictive validity and actionable clinical benefit. Placement of a study within one of these stages was not interpreted as a formal quality grade. Detailed characteristics of the representative studies summarized in Tables 1–5, including sample size, cohort source, study design, AI method, input modality, outcome definition, validation approach, and main performance metrics, are provided in Supplementary Material 4. Evidence was weighted according to study design, sample size, risk of data leakage, outcome definition, validation strategy, calibration, comparator performance, and clinical relevance. Consequently, a technically strong cross-sectional study could provide more reliable measurement evidence than a small or poorly validated longitudinal model, even though the latter was closer to a clinical outcome.

**TABLE 1:** Evidence boundaries for imaging AI in KOA structural phenotyping, tissue segmentation and severity recognition.

References	AI aim and method	Main finding	Recommended use and evidence boundary
Dang et al. (2026)	Nested case-control OAI study; MRI radiomics assessed thigh muscle quality and predicted incident KOA	Extensor radiomics score best predicted incident KOA within 4 years and outperformed extensor cross-sectional area, intramuscular fat, flexor/accessory muscle models and clinical models. Combining other features did not add significant value	Longitudinal risk-marker evidence. Supports inclusion of muscle and soft-tissue phenotypes in KOA stratification, but external validation and intervention-response validation are needed
Yao et al. (2026)	Retrospective two-center conventional MRI cohort comparing 3D Res U-net, 3D U-net and 3D V-net	3D Res U-net outperformed other models in internal and external validation. Dice values for lateral tibiofemoral, medial tibiofemoral and patellofemoral cartilage were higher internally than externally, with lower boundary error	Cross-center segmentation-validation evidence. Can support cartilage thickness, volume and radiomics extraction, but error propagation still requires analysis
Mu et al. (2026)	OAI cohort; nnU-Net segmented 29 knee structures, extracted radiomic features and built a KOA risk model	The final model integrated 129 radiomic features. It predicted 4-year incident KOA with AUC 0.918 and improved young orthopedic surgeons’ accuracy from 0.605 to 0.813	Imaging risk-atlas evidence. Potential expert-assistance tool, but independent external validation and workflow evaluation are needed
Li et al. (2026)	OAI DESS and in-house FSE MRI data; ERANet semi-supervised meniscus segmentation under low-annotation settings	ERANet performed well across two MRI sequence types and annotation ratios, improving segmentation with limited labels	Low-label segmentation evidence. Useful for reducing annotation burden, but clinical value depends on downstream risk prediction or decision gain
Lau et al. (2025)	Retrospective MOST cohort; diffusion model generated follow-up patellar shape and SynPatNet predicted patellofemoral OA, tibiofemoral OA and knee replacement	The diffusion model simulated 60-month patellar deformation and osteophyte formation. Synthetic follow-up shape improved prediction of patellofemoral OA and replacement, but had limited value for tibiofemoral OA onset	Shape-generation and prognostic evidence. May complement patellofemoral phenotyping, but links to pain, function and treatment action need clarification
Chavoshi et al. (2026)	OAI cohort; ResNet18 and ConvNeXt compared with BiomedCLIP and RAD-DINO embeddings plus XGBoost for radiographic feature classification	Supervised DL outperformed foundation-model embeddings across tasks. ConvNeXt performed best for KL grading. Text embeddings gave only small gains, and BiomedCLIP zero-shot knee-film recognition accuracy was 91.14%	Model-comparison evidence. Foundation embeddings are attractive but should not be assumed superior to supervised task-specific models. Failure cases and interpretability should be reported
Park et al. (2026)	Swin-UNet cGAN for MRI cartilage segmentation with development and validation	The model improved extraction of knee-cartilage contours, but external-validation performance declined relative to internal data	Segmentation evidence. Provides a basis for structural phenotyping, but uncertainty, cross-device stability and error propagation should be reported

AI, artificial intelligence; AUC, area under the receiver operating characteristic curve; cGAN, conditional generative adversarial network; DESS, dual-echo steady-state; DL, deep learning; FSE, fast spin echo; KL, Kellgren-Lawrence; KOA, knee osteoarthritis; MOST, multicenter osteoarthritis study; MRI, magnetic resonance imaging; OA, osteoarthritis; OAI, osteoarthritis initiative; 3D, three-dimensional.

**TABLE 2:** Evidence for gait AI, wearable sensing and real-world KOA load assessment.

References	Method/device	Main outcome	Performance, limitation or recommendation
Mapinduzi et al. (2026)	Twenty KOA patients and 20 healthy controls; questionnaires, clinical functional tests, smart insoles and 7-day wearable monitoring integrated into a hybrid digital phenotyping framework	The framework stably integrated multisource data and distinguished patients from controls. KOA patients showed slower speed, shorter stride length and reduced propulsive gait. Speed, stride length, cadence and loading phase were negatively correlated with KOA status	Feasibility evidence for real-world digital phenotyping. Small sample; mainly supports monitoring framework. Longitudinal outcomes, adherence and alert thresholds require validation
Snyder et al. (2025a)	Forty-seven participants completed walking, running, sit-to-stand and stand-to-sit tasks. Custom instrumented insole data were input into recurrent neural networks to predict MJCF, KFM and KAM waveforms	Load estimation was extended from straight walking to activities closer to daily life. Most activity-specific load waveforms showed strong correlations, but sit-to-stand and stand-to-sit KAM showed only moderate correlations	Technical evidence for load estimation. KAM predictability varies by task, and straight-walking performance should not be generalized to all daily activities
Feng et al. (2025)	Four IMUs and six EMG sensors collected walking data from 18 valid participants. A self-aware IMU-EMG fusion model with attention dependence estimated KAM during walking	The model reduced relative root-mean-square KAM error by 9.15%, suggesting that fusion of muscle electrical activity and inertial signals improves dynamic load estimation	Biomechanical-target estimation evidence. Deployment depends on sensor placement, calibration, signal quality and patient wearing adherence
Moura et al. (2025)	Three-dimensional gait kinematic time series analyzed using deep neural networks and transfer learning for KOA classification or prediction	Continuous gait waveforms provided discriminative information beyond traditional discrete biomechanical variables	Methodological evidence. Limited by laboratory acquisition; ecological validity and cross-center generalizability remain to be validated

AI, artificial intelligence; EMG, electromyography; IMU, inertial measurement unit; KAM, knee adduction moment; KFM, knee flexion moment; KOA, knee osteoarthritis; MJCF, medial tibiofemoral joint contact force.

**TABLE 3:** AI-enabled computational pipelines, biomechanical modeling, and digital-twin-oriented evidence in KOA.

References	Design and data	Main finding	Interpretation and recommendation
Paz et al. (2024)	OAI cohort; template FEA model; neural network estimation of peak knee contact force and compartmental load distribution	NN-estimated load inputs improved FE model classification of OA onset or severity at 8 years (AUC about 0.70). Adding individual cartilage thickness did not further improve classification	Longitudinal prediction and mechanistic-modeling evidence. Supports AI-FEA for load-related risk stratification, but remains limited by template models, load-input estimation and untested clinical utility
Hoyer et al. (2025)	OAI qMRI cohort; DL segmentation of knee tissues, extraction of cartilage thickness, T2 relaxation and bone/meniscal morphology; propensity matching and elastic-net regression	Cartilage-thickness PC2 was protective for OA onset and knee replacement. Femoral T2 PC7, tibial T2 PC8 and medial meniscus PC10 were linked to higher OA risk. In replacement analyses, tibial bone-shape PC9 increased risk, whereas femoral and tibial thickness PCs were protective	Tissue phenotype and digital-twin-oriented feature-space evidence. Supports mechanistic hypotheses and long-term risk stratification, but prospective decision-utility validation is still needed
Jayakumar et al. (2025)	Single-center randomized trial comparing an AI-enabled decision aid using a digital-twin framework with education alone in KOA/TKA shared decision-making	The intervention group had higher K-DQI decision-quality scores, lower decisional conflict, higher KOOS-JR at 6–9 months and greater concordance between treatment choice and preference	Clinical decision-utility evidence with relatively high translational relevance. Multicenter, blinded or real-world implementation studies are still needed to assess generalizability and implementation effectiveness
Lan et al. (2024)	Sixty primary TKA patients, with 30 in an AI-based 3D preoperative planning group and 30 in a 2D radiography group; component size and lower-limb alignment planning were compared	The AI group had higher femoral and tibial component-size prediction accuracy than the 2D group, and VCA and HKA prediction were also more accurate	Perioperative feasibility evidence. Supports standardized preoperative planning, but effects on long-term function, revision rate and resource use remain untested

AI, artificial intelligence; AUC, area under the receiver operating characteristic curve; DL, deep learning; FE, finite element; FEA, finite element analysis; HKA, hip-knee-ankle angle; K-DQI, knee osteoarthritis decision quality instrument; KOA, knee osteoarthritis; KOOS-JR, knee injury and osteoarthritis outcome score for joint replacement; NN, neural network; OA, osteoarthritis; OAI, osteoarthritis initiative; PC, principal component; qMRI, quantitative magnetic resonance imaging; TKA, total knee arthroplasty; VCA, femoral valgus correction angle; 2D, two-dimensional; 3D, three-dimensional.

**TABLE 4:** Representative evidence for KOA progression prediction, risk phenotypes, candidate endotypes and treatment-response prediction.

References	Evidence type/method and data	Main finding	Recommended use and evidence boundary
Zhou et al. (2023)	FNIH cohort development and BMF validation; UPLC-MS/MS quantification of serum peptides; bootstrap elastic net and backward elimination for biomarker selection; prediction of 48-month joint-space loss and pain progression	A model comprising 15 serum peptide biomarkers corresponding to 13 proteins outperformed baseline KL grade, pain score and urinary biomarkers	Molecular prediction evidence. Suggests that blood-based systemic molecular signals may predict both structural progression and pain worsening. Cost, accessibility and incremental decision value require evaluation
Alkhatatbeh et al. (2026)	OAI cohort; clinical features compared with DESS-MRI radiomics for predicting response to hyaluronic acid injection; different cross-validation strategies assessed	The clinical model performed best and outperformed radiomics. Validation split by knee rather than participant inflated combined-model AUC, indicating leakage from bilateral knees	Methodological warning evidence. Radiomics must be compared with simple clinical models and must avoid leakage from two knees of the same participant
Rockel et al. (2025)	Multi-fluid multi-omics in replacement patients; omicVAE and K-means identified endotypes; ML models predicted 1-year WOMAC pain and functional response	Three candidate molecular endotypes were identified. Integrated multi-omics and clinical data predicted pain and function response better than any single data domain	Endotype exploratory evidence. More suitable for hypothesis generation and trial stratification than direct routine prescription
Lee et al. (2025)	OAI development and MOST external validation; predicted radiographic progression in KL 0–2 knees over 4–5 years	The final model used accessible variables, including baseline KL grade, contralateral knee OA, lateral joint-space narrowing, BMI, medial joint-space narrowing and total WOMAC score	Externally validated prediction evidence. The model is simple and actionable, but calibration, decision curves and clinical-impact evaluation remain needed

AUC, area under the receiver operating characteristic curve; BMF, biomarker factory; BMI, body mass index; DESS, dual-echo steady-state; FNIH, foundation for the national institutes of health; KL, Kellgren-Lawrence; ML, machine learning; MOST, multicenter osteoarthritis study; MRI, magnetic resonance imaging; OA, osteoarthritis; OAI, osteoarthritis initiative; UPLC-MS/MS, ultra-performance liquid chromatography-tandem mass spectrometry; WOMAC, Western Ontario and McMaster Universities Osteoarthritis Index.

**TABLE 5:** Evidence relevant to KOA AI clinical translation, rehabilitation feedback, and implementation.

References	Aim and method	Main finding	Translational meaning and evidence boundary
Wang et al. (2021)	Randomized gait-retraining study; shoe-mounted IMUs and an artificial neural network provided real-time KAM feedback; individualized retraining compared with ordinary walking training in early medial KOA	After retraining, foot progression angle decreased by 6.3° on average, first-peak KAM decreased, and VAS pain and KOOS improved. Second-peak KAM, KFM and KAAI did not show corresponding reductions	Load-targeted rehabilitation intervention evidence. Shows that AI-estimated KAM can be translated into prescription feedback; longer follow-up and real-world validation are needed
Panfilov et al. (2025)	OAI cohort; CNN plus transformer performed cross-slice and cross-modal fusion to assess radiography, MRI and quantitative T2 maps for predicting imaging progression over different time windows	Structural MRI alone predicted 2–8-year progression stably and was not inferior overall to multimodal fusion. Multimodal models mainly benefited 1-year short-term prediction. Prediction was more accurate in patients with prior knee injury or surgery	Multimodal prediction evidence. Additional modalities do not necessarily improve performance; modality ablation and time-window analyses are needed
York et al. (2024)	Retrospective cohort study; routine knee radiograph AI tool automatically identified candidates for partial knee arthroplasty (PKA)	EfficientNet-ES identified PKA candidates with AUC 0.95 and accuracy 79%–80%, but was weaker for identifying TKA or patellofemoral replacement indications	Preoperative triage evidence. May assist PKA candidate screening, but cannot replace integrated assessment of history, examination and patient preference
van Helvoort et al. (2020)	IMI-APPROACH cohort; clinical, imaging, biochemical and omics variables integrated to identify progression-prone KOA	The machine-learning selection process was feasible. Selected participants had more pain, stiffness and functional impairment than non-selected participants and higher predicted pain and structural progression risk	Cohort-resource and implementation-foundation evidence. Supports external validation and subtype research

AI, artificial intelligence; AUC, area under the receiver operating characteristic curve; CNN, convolutional neural network; IMI-APPROACH, Applied Public-Private Research enabling OsteoArthritis Clinical Headway; IMU, inertial measurement unit; KAAI, knee adduction angular impulse; KAM, knee adduction moment; KFM, knee flexion moment; KOA, knee osteoarthritis; KOOS, knee injury and osteoarthritis outcome score; MRI, magnetic resonance imaging; OAI, osteoarthritis initiative; PKA, partial knee arthroplasty; TKA, total knee arthroplasty; VAS, visual analog scale.

### 2.4 Methodological quality and risk-of-bias considerations

A formal study-level risk-of-bias assessment using QUADAS-2, PROBAST, the Newcastle–Ottawa Scale, or ROBINS-I was not performed. This decision reflected both the narrative design of the review and the heterogeneity of the included evidence, which encompassed diagnostic-accuracy studies, prognostic models, longitudinal observational studies, image-segmentation and classification studies, wearable-sensor engineering studies, computational biomechanical simulations, rehabilitation studies, and perioperative applications. No single assessment instrument was applicable across these designs, and the purposive rather than exhaustive selection of representative studies would have limited the interpretability of pooled risk-of-bias ratings.

Instead, studies discussed as core evidence were critically interpreted using a common set of methodological domains: sample size and cohort representativeness; independence of training, validation, and test data; participant-level rather than knee-level or image-level data partitioning; risk of information leakage; clarity of the reference standard or outcome definition; handling of missing data and class imbalance; internal and external validation; calibration and decision-analytic evaluation where applicable; comparison with simple clinical or conventional models; reporting of uncertainty and failure cases; reproducibility; and the extent to which the reported output could be linked to a defined clinical action.

This domain-based appraisal was used to guide the narrative weighting and wording of conclusions rather than to generate a numerical quality score or formal certainty-of-evidence grade. Cross-sectional findings were interpreted as associations or phenotype-identification evidence rather than causal evidence. Internally validated models were not considered evidence of generalizability, and technical performance was not interpreted as clinical utility in the absence of prospective, external, or implementation-level evaluation.

## 3 Biomechanical basis of KOA

The biomechanics of KOA can be understood at three levels. The first is external load input, including body weight, lower-limb alignment, ground reaction force, plantar pressure and movement pattern. The second is tissue tolerance, determined by the quality and functional state of cartilage, menisci, subchondral bone, synovium, the infrapatellar fat pad and muscle. The third is behavioral feedback, including pain perception, fear of falling, movement avoidance and rehabilitation adherence. These levels amplify one another through a mechanical-biological-behavioral loop and contribute to the marked clinical heterogeneity of KOA (Figure 3). AI studies that ignore any one level may yield models that appear accurate but have limited clinical interpretability.

**FIGURE 3:**
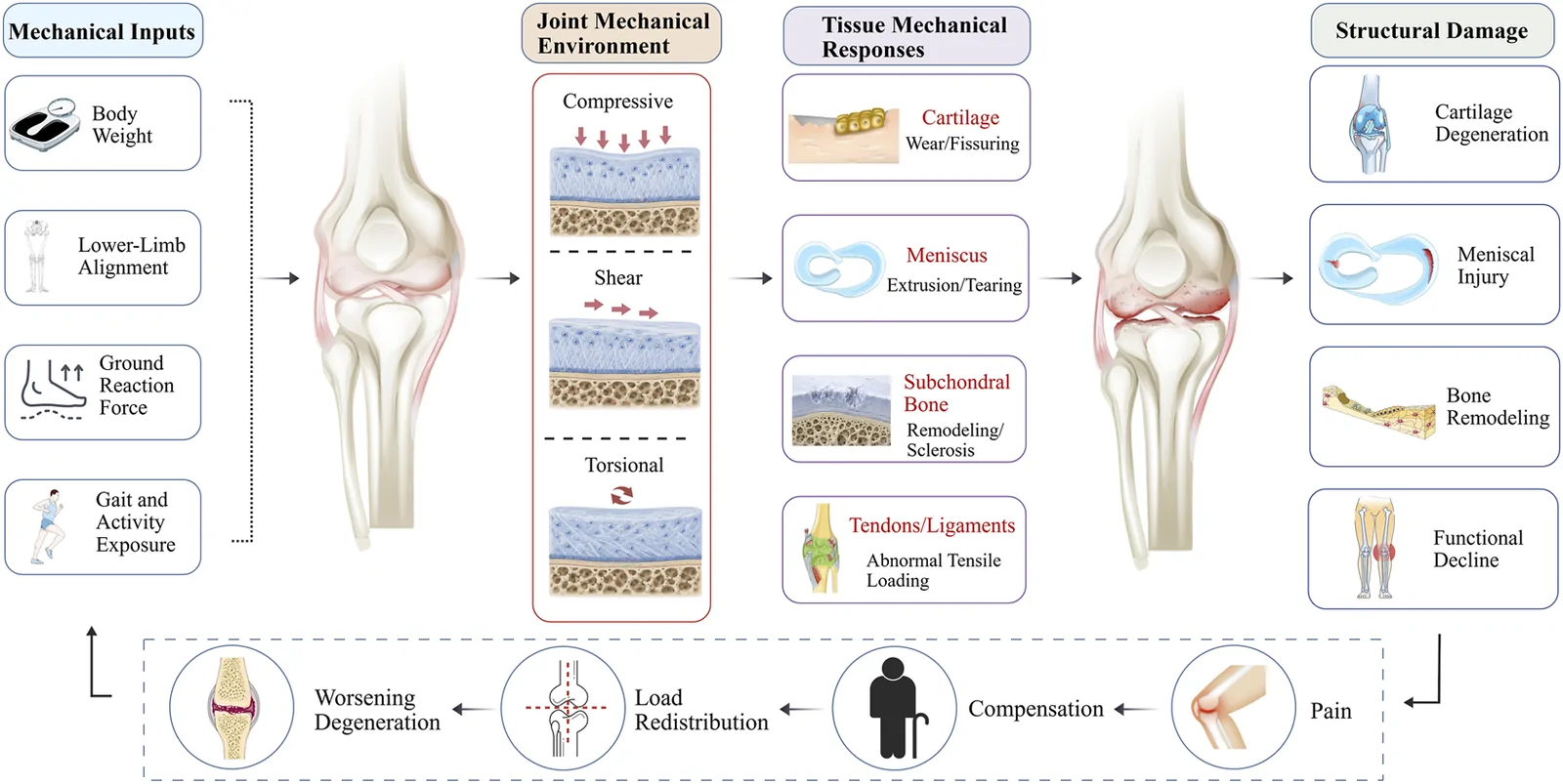
Mechanical, tissue and behavioral feedback mechanisms in knee osteoarthritis onset and progression. External mechanical inputs—including body weight, lower-limb alignment, ground-reaction force, gait strategy and cumulative activity exposure—alter the joint mechanical environment. Compression, shear, torsion and repeated impact interact with the tolerance of cartilage, menisci, subchondral bone, synovium, ligaments and periarticular muscle. Tissue damage and inflammation may generate pain, movement avoidance, compensatory gait and redistribution of joint loading, thereby forming a mechanical–biological–behavioral feedback loop. Longitudinal studies integrating gait, activity and cartilage damage support the importance of combined loading and structural vulnerability, whereas imaging-radiomics studies suggest that muscle quality may contribute additional prognostic information (Costello et al., 2023; Dang et al., 2026). The arrows represent biologically plausible and mutually reinforcing relationships rather than proof of a single linear causal pathway. AI may help quantify components of this system, but causal interpretation requires longitudinal, mechanistic or interventional evidence.

### 3.1 Multidimensional properties of mechanical loading

Mechanical loading is a central biomechanical variable in KOA onset and progression. It is not a single peak value but a multidimensional and time-varying exposure determined jointly by direction, magnitude, frequency, cumulative exposure and tissue tolerance. Moderate dynamic mechanical stimulation helps maintain cartilage matrix homeostasis. By contrast, chronic abnormal compression, shear and impact can shift chondrocytes from anabolic matrix maintenance toward catabolism mediated by matrix metalloproteinases, ADAMTS and inflammatory mediators. This process is amplified by subchondral bone remodeling and synovitis (Hodgkinson et al., 2022). Obesity is not simply a mechanical load. It increases axial load across the tibiofemoral joint, but it also reduces tissue mechanical tolerance through adipose tissue dysfunction, dysregulated adipokine secretion and low-grade inflammation (Binvignat et al., 2024). In people with obesity and KOA, a randomized trial of once-weekly semaglutide supports the possibility that metabolic intervention can improve body weight and pain-related outcomes (Bliddal et al., 2024). It is used here as background evidence that metabolic-inflammatory burden and mechanical loading may act together, suggesting that treatment strategies may integrate load reduction with inflammatory and metabolic modulation.

Biomechanical research should therefore avoid equating higher load with higher risk. The same knee adduction moment (KAM) or contact pressure may have different tissue consequences across patients with different cartilage quality, meniscal integrity, bone density, muscle control and inflammatory states. The value of FEA and musculoskeletal modeling lies in their ability to provide tissue-level interpretations of stress. Their conclusions nevertheless require cross-validation against clinical imaging and functional outcomes, to avoid mistaking modeling assumptions for clinical facts (Xie et al., 2023; Guo et al., 2025).

### 3.2 Static alignment and dynamic exposure

Alignment parameters and gait features link static imaging structure to real dynamic loading. Deep learning-based automated measurement of full-length lower-limb radiographs can efficiently obtain the hip-knee-ankle angle (HKA), mechanical lateral distal femoral angle (mLDFA) and medial proximal tibial angle (MPTA), while reducing variability in manual measurement (Lee H. S. et al., 2024; Yang et al., 2024). Static alignment, however, only reflects the anatomical basis of load shift and cannot replace assessment of dynamic exposure. The effects of foot progression angle, step width and walking speed on KAM vary substantially across individuals. Individualized gait modification should therefore not require all patients to adopt a uniform internally or externally rotated pattern. Instead, it should estimate the response curve between gait change and medial compartment loading for each patient, and then select an adjustment that lowers peak KAM without inducing compensatory moments (Kwon et al., 2019; Baghi et al., 2025a; Baghi et al., 2025b). Customized instrumented insoles can estimate medial tibiofemoral joint contact force (MJCF), knee flexion moment (KFM) and KAM waveforms during walking, running and sit-to-stand transitions. Most load waveforms show strong correlations, but KAM estimation during tasks with different loading patterns or lower KAM amplitudes, such as sit-to-stand, shows greater error variability (Snyder et al., 2025a). Self-aware models that fuse IMU and electromyography (EMG) signals further indicate that multisource wearable signals can reduce KAM estimation error, providing a technical basis for monitoring dynamic load outside the laboratory (Feng et al., 2025).

Real-world mechanical exposure adds further complexity. Straight-line walking in the laboratory cannot fully represent stair negotiation, turning, long-distance walking or load strategies during pain fluctuations. Longitudinal machine learning work in the MOST cohort incorporated gait, physical activity and tibiofemoral cartilage damage within a single analytical framework. The results indicated that risk is not determined by step count alone, but by the combination of activity dose, impact or support strategy, and pre-existing structural vulnerability (Costello et al., 2023; Bacon et al., 2026). Non-radiographic monitoring has also shown that plantar pressure and trunk-pelvis motion variables can distinguish participants with KOA from controls. A decision-tree model achieved an accuracy of 89.3% and a sensitivity of 85.7%, suggesting that kinetic-chain variables away from the knee may also carry discriminative information associated with KOA status (Sarmah et al., 2024).

### 3.3 Muscle, pain and functional feedback

Muscle quality and pain behavior further explain the mismatch between structural damage and functional decline. In 448 OAI participants, Dang et al. found that a higher baseline radiomics score for quadriceps extensor muscle, indicating poorer extensor quality, was associated with higher future KOA risk. Test-set AUC was 0.832, outperforming extensor cross-sectional area (AUC 0.595), intramuscular fat area (AUC 0.600), radiomics scores for flexor and accessory muscle groups, and clinical models (Dang et al., 2026). After AI-based CT muscle segmentation in 79 patients with end-stage medial KOA, Suzuki et al. found that greater varus HKA was associated with lower mean CT values in the gluteus maximus, gluteus medius/minimus, adductors, quadriceps and posterior calf muscles, suggesting more severe fatty degeneration. HKA was not significantly associated with standardized muscle volume (Suzuki et al., 2025).

For pain, machine learning and class activation mapping analyses by Zhao et al. showed that effusion-synovitis and cartilage loss accounted for 30.9% and 30.6% of structural abnormalities associated with pain severity, respectively, whereas meniscal injury accounted for 23.5%. Model AUC was only about 0.67–0.70, indicating that structural lesions explain part of the pain variance but should not be interpreted as a single causal mechanism (Zhao et al., 2024). Pain can also alter load exposure through activity avoidance, gait compensation and rehabilitation adherence. It may thereby create a closed feedback loop among structural lesions, pain perception and behavioral response (Bacon et al., 2022; Bacon et al., 2024).

## 4 AI-enabled multimodal evidence pathways

The multimodal evidence chain for AI in KOA can be grouped into imaging data, movement data, wearable-sensor data and computational-simulation data. Imaging data capture tissue anatomy and morphological change. Movement data reflect functional performance under loaded conditions. Wearable-sensor data capture cumulative mechanical exposure in daily life. Computational simulation provides model-based estimates of joint contact force and tissue mechanical response. These data streams address different clinical questions. Information fusion may generate complementary evidence, but it may also introduce measurement error, sampling bias and interpretive limits arising from cross-modal coupling.

### 4.1 Imaging AI, from grading to tissue phenotyping

Imaging AI has the most mature evidence base in KOA, but reporting should specify which tissue structures can be quantified and how accurately they can be measured (Table 1). Cartilage remains the most frequently assessed MRI biomarker. Effusion-synovitis is well suited to automated assessment because of its high T2-signal contrast, and meniscal tear detection is also relatively mature. By contrast, cartilage loss has been studied most extensively, but remains difficult to localize and quantify consistently because cartilage boundaries are thin, focal defects are common and performance varies across imaging sequences (McDonald et al., 2025). Reviews of deep learning in radiography and MRI show that automated grading, segmentation, lesion detection and progression prediction now form a relatively complete methodological spectrum. However, insufficient external validation, inconsistent annotation standards and limited transferability across scanners and sequences remain major barriers to clinical use (Ebrahimkhani et al., 2020; Yeoh et al., 2021; Nevalainen et al., 2026; Rani et al., 2026). MRI deep learning studies on contrast synthesis are therefore better viewed as imaging preprocessing and feasibility evidence, rather than core evidence for KOA clinical outcomes.

The value of recent imaging models lies not only in improved classification accuracy, but also in expanding the range of visible structures that can be analyzed. Yao et al. compared 3D Res U-net, 3D U-net and 3D V-net in 254 conventional proton-density-weighted fat-suppressed MRI scans. In the external validation set, 3D Res U-net achieved Dice coefficients of 0.846, 0.837 and 0.819 for lateral tibiofemoral, medial tibiofemoral and patellofemoral cartilage, respectively. These findings suggest that robust automated cartilage segmentation may be feasible from routine clinical sequences, although external performance was lower than internal validation and cross-center calibration remains necessary (Yao et al., 2026). Whole-knee MRI radiomic atlases (Mu et al., 2026), semi-supervised meniscus segmentation (Li et al., 2026) and diffusion models of patellar morphology (Lau et al., 2025) further convert cartilage, menisci, bone shape and patellofemoral structures into computable features for disease-risk or outcome prediction. Foundation-model embedding studies indicate that supervised deep learning still outperforms general foundation models for fine-grained feature classification on knee radiographs (Chavoshi et al., 2026). Segmentation-error studies further show that some radiomics classifiers are robust to perturbations of cartilage masks, whereas tree-based models are more sensitive to dilation errors. Models should therefore report Dice coefficients, surface distances, external validation results and error-propagation analyses (Bercaw et al., 2025; Fox et al., 2026).

### 4.2 Gait AI and wearable sensing

Gait AI and wearable sensing help address the limited biomechanical information provided by static imaging (Table 2). Deep neural networks and transfer learning can identify KOA from gait kinematic time-series data, whereas vision-based gait models extend markerless motion capture toward low-cost screening settings (Ali et al., 2025; Kaya et al., 2025; Moura et al., 2025). Unlike simple classification tasks, load-estimation studies are closer to clinical needs. Pressure-sensing insoles combined with neural networks can estimate medial tibiofemoral joint contact force, knee flexion moment and knee adduction moment (Snyder et al., 2025a). IMU-EMG fusion models can reduce estimation error for knee adduction moment (Feng et al., 2025). Plantar-pressure analysis combined with trunk-pelvis motion can distinguish people with KOA from healthy individuals without radiographic assessment (Sarmah et al., 2024). Dynamic gait parameters obtained from preoperative wearable sensors can also predict functional recovery after total knee arthroplasty (TKA), suggesting that gait features may serve both as diagnostic signals and as early indicators of postoperative recovery trajectories (Kluge et al., 2018; Lee D. W. et al., 2024).

Sensor-based studies need to balance technical feasibility with clinical usability. Additional sensors often improve model performance, but real-world deployment is constrained by cost, wear adherence, calibration procedures and missing data. Some small-sample or engineering-implementation studies, including TinyML-based wearable systems for early screening, are useful references for device deployment and edge-computing methods. However, they should not be treated as core evidence that already supports clinical diagnostic or therapeutic utility (Bharanidivya and Dhanalakshmi, 2026). Studies optimizing sensor placement indicate that ground-reaction-force estimation and gait-event detection can be achieved with fewer IMU combinations (Havashinezhadian et al., 2023; Aragón-Basanta et al., 2025). Work on knee-kinematics prediction further emphasizes that future systems should prespecify a minimal sensor configuration, calibration protocol and data-quality threshold, rather than indiscriminately collecting all available signals (Tan et al., 2022).

### 4.3 Computational simulation and digital twins

AI-enabled computational pipelines can help move data-driven modeling toward mechanism-constrained analysis, whereas conventional FEA and musculoskeletal models provide complementary mechanistic context (Table 3). Paz et al. built template FEA models for 97 knees from the OAI cohort and simulated cartilage degeneration under three gait-loading conditions (Paz et al., 2024). After incorporating neural network-estimated peak joint contact force and compartmental load sharing, the model distinguished knees that developed mild-to-moderate osteoarthritis (OA) over 8 years from those that did not, with an area under the receiver operating characteristic curve (AUC) of 0.70 (p < 0.05). By contrast, adding individual femoral and tibial cartilage thickness did not improve classification performance. This finding suggests that individualized modeling does not necessarily benefit from adding more variables, and that the most discriminative information may lie in the appropriate balance between loading, geometry and material assumptions.

FEA studies of UKA (Liu et al., 2024) and TKA (Ou et al., 2024; Zhang et al., 2026) further show that bone density, alignment parameters and implant layout can substantially alter postoperative stress distribution. Quantitative MRI (qMRI)-based digital-twin-oriented studies compress cartilage thickness, T2 values, bone shape and meniscal morphology into interpretable feature spaces. Within these spaces, femoral cartilage thickness repeatedly appears as a protective marker in analyses of OA onset and joint replacement (Hoyer et al., 2025). The potential value of digital-twin-oriented frameworks should therefore not be limited to generating complex three-dimensional models. Rather, such frameworks may link modifiable mechanical parameters with tissue metrics that can be tracked over time (Jayakumar et al., 2025).

## 5 AI-enabled biomechanical phenotyping, risk phenotypes and candidate modifiable factors

The value of AI in KOA research does not lie in directly proving causality. Rather, it lies in identifying reproducibly measurable phenotypes from high-dimensional imaging, gait, sensor, molecular and clinical data that are associated with disease susceptibility, progression, symptoms or treatment response. Such findings can be divided into three categories: statistically associated risk markers, mechanistically plausible biomechanical hypotheses and candidate modifiable factors that may inform clinical decision-making (Figure 4). Radiomics, deep learning and clustering models can reveal complex multivariable associations. However, these associations should be interpreted cautiously as disease drivers or therapeutic targets only after further validation in longitudinal follow-up, intervention-response studies or mechanistic models. To avoid conflating descriptive phenotypic patterns with longitudinal risk markers, treatment-response patterns, mechanistically defined endotypes, or modifiable intervention targets, we apply the following terminology consistently throughout this review.

**FIGURE 4:**
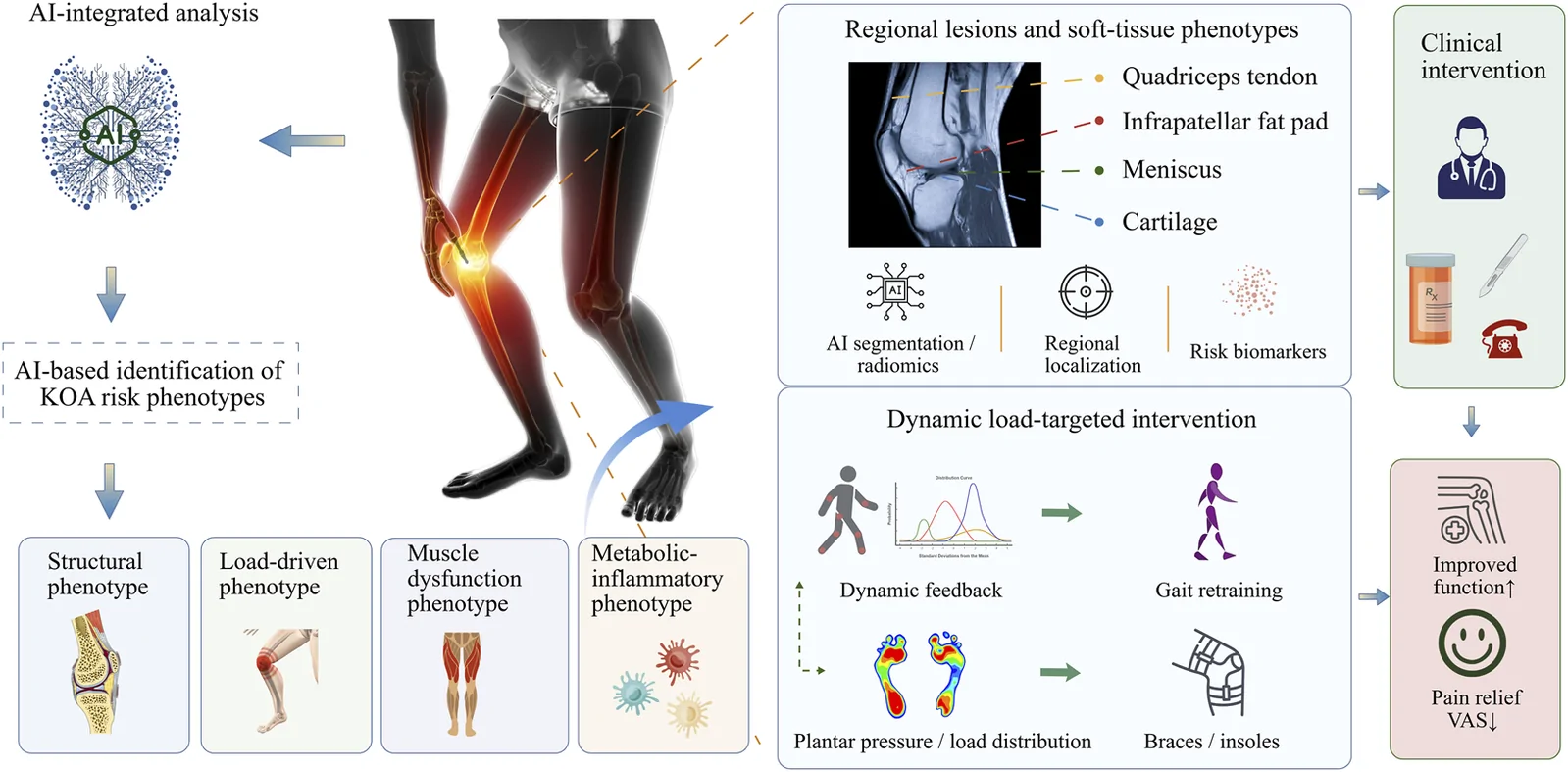
Conceptual decision pathway from AI-derived knee osteoarthritis phenotypes to candidate clinical actions. AI may integrate imaging, gait, wearable-sensor and clinical data to identify structural, biomechanical-loading, muscle-dysfunction, digital-behavioral and metabolic-inflammatory phenotypes. These patterns should be termed risk phenotypes only when their association with a future outcome has been demonstrated longitudinally. Potential decision pathways include: regional structural abnormalities prompting targeted imaging review, orthopedic assessment or surgical-planning evaluation; abnormal loading patterns prompting gait retraining, brace or insole assessment and load-monitoring; muscle-dysfunction patterns prompting individualized strengthening and functional rehabilitation; digital-behavioral patterns prompting adherence support or adjustment of follow-up intensity; and metabolic-inflammatory patterns prompting weight-management or medical assessment.

Terminology glossary. To ensure consistent interpretation throughout this review, the following terms are used according to predefined operational meanings. Phenotype is used as an umbrella term for a reproducibly measurable pattern of structural, biomechanical, molecular, symptomatic, functional, or digitally derived features, without implying an underlying causal mechanism. Biomechanical phenotype refers specifically to a reproducible pattern of joint loading, movement, alignment, muscle function, or other mechanically relevant characteristics. Risk phenotype is reserved for a phenotype that has been shown in longitudinal data to be associated with a defined future outcome, such as KOA onset, structural progression, pain progression, or functional decline. Cross-sectional associations alone are therefore described as phenotypic features, associated markers, or candidate risk markers rather than established risk phenotypes. Treatment-response phenotype refers to a baseline or early-change pattern associated with differential response to a defined intervention and is distinguished from a general prognostic marker that predicts outcome irrespective of treatment. Endotype is reserved for a reproducible subgroup characterized by a biologically or mechanistically coherent underlying pathway with demonstrated longitudinal or treatment relevance; statistically derived clusters without sufficient mechanistic validation are referred to as candidate endotypes. The term endophenotype is not used as a separate category because its meaning is narrower and inconsistently applied in the KOA literature. Finally, a candidate modifiable factor denotes a phenotypic or biomechanical feature that could plausibly be altered by a clinically feasible intervention, whereas the term intervention target is reserved for factors for which modification has been shown to improve patient-relevant outcomes.

### 5.1 Regional lesions and soft-tissue phenotypes

The clinical value of structural lesions lies in regional localization, not merely in global grading. Multilabel segmentation (Yu et al., 2026), meniscus segmentation (Li et al., 2026) and patellar shape analysis (Lau et al., 2025) suggest that AI can extract tissue location, extent and shape with greater granularity than conventional grading. Radiomics studies further suggest that the direction of risk depends on tissue and feature type. Subchondral bone, cartilage, menisci and whole-knee atlas features may improve early recognition or progression stratification, but risk weights differ across regions. It is therefore unsafe to generalize that radiomics is universally associated with KOA risk (Fox et al., 2026; Mu et al., 2026). Muscle and fat-pad studies similarly show that non-cartilaginous soft tissues are not merely background variables. Reduced extensor quality can predict future KOA risk, and infrapatellar fat-pad radiomics may reflect the risk of structural abnormalities (Consolini et al., 2025).

Regional structural abnormality does not necessarily imply a directly modifiable target. Cartilage defects, meniscal injury and bone marrow lesions may result from abnormal loading, but they may also change load transmission and create a bidirectional relationship. These distinct evidentiary roles should therefore be explicitly distinguished. Cross-sectional studies support correlation as a marker. Longitudinal cohorts support predictive value. Evidence that modifying the candidate factor changes patient-relevant outcomes is needed before it can be regarded as a clinically actionable intervention target. This distinction helps prevent overinterpretation of high-dimensional AI associations as etiological evidence.

### 5.2 Biomechanical phenotypes and candidate modifiable factors

Dynamic loading variables, including the knee adduction moment, joint contact force and plantar-pressure distribution, are described here as biomechanical phenotypes when they characterize measurable movement or loading patterns. They are described as candidate modifiable factors when a clinically feasible intervention could plausibly alter them. The term intervention target is reserved for factors for which modification has been shown to improve patient-relevant outcomes. Dynamic loading represents a plausible mechanistic link between structural progression and symptom fluctuation and may therefore provide a more modifiable biomechanical target than imaging grade alone. A 6-week sensor-driven gait-retraining study provides direct intervention evidence for sensor-guided biomechanical feedback. Compared with ordinary walking training, real-time KAM feedback reduced the first KAM peak by about 16.5% in patients with early medial KOA, while visual analog scale pain decreased and Knee injury and Osteoarthritis Outcome Score (KOOS) improved. The second KAM peak, KFM and knee adduction angular impulse did not show corresponding reductions, and adverse-event data were not reported (Wang et al., 2021). These findings suggest that load-targeted exercise prescription is feasible, but longer follow-up, real-world adherence assessment and patient-centered outcome validation are still needed.

### 5.3 Treatment-response prediction and candidate treatment-response phenotypes

Treatment-response prediction should be distinguished from general prognosis and risk stratification. In this review, a treatment-response phenotype refers only to a baseline or early-change pattern associated with differential benefit from a defined intervention. Biomarkers associated with future structural or pain progression, but not with differential treatment effects, are classified as prognostic markers or risk phenotypes rather than treatment-response phenotypes.

Current KOA studies provide preliminary evidence for treatment-response prediction, but relatively few establish a reproducible treatment-by-phenotype interaction. Treatment-allocation analyses of diet and exercise, prediction of response to hyaluronic acid injection, and post-arthroplasty outcome modeling are therefore interpreted as candidate treatment-response evidence rather than as established treatment-response phenotypes.

Blood molecular features may complement imaging and clinical variables for predicting concurrent structural and pain progression and are therefore interpreted as prognostic biomarkers or candidate risk-phenotype components rather than treatment-response phenotypes (Zhou et al., 2023). Treatment-allocation studies also caution that mechanism-related variables do not necessarily improve practical prescription decisions. In a precision-medicine analysis of the IDEA diet and exercise trial, an optimal individualized treatment rule that incorporated baseline gait force variables was not significantly better than fixed treatment allocation. For most clinical outcomes, combined diet and exercise remained the optimal strategy. Diet alone was optimal only for a small number of specific outcomes, such as changes in interleukin-6 or compressive force (Kostic et al., 2025). A study predicting response to hyaluronic acid injection provides an even clearer methodological caution (Alkhatatbeh et al., 2026). When cross-validation was grouped strictly by participant, the clinical model achieved an AUC of 0.716, whereas a DESS-MRI radiomics model was close to random (AUC 0.499), and the combined model did not outperform clinical variables. When training and validation were split by knee rather than participant, the AUC was inflated to 0.773. Validation design therefore directly determines the credibility of radiomics conclusions.

Multimodal stratification can help explain disease heterogeneity, but it cannot replace causal inference. Machine learning analyses of single-leg standing and step-up or step-down kinematics in early KOA show that functional tasks can reveal biomechanical movement phenotypes that are not fully captured by static imaging (Hwang et al., 2025b; Hwang et al., 2025a). Multi-omics clustering and post-arthroplasty response classification suggest that candidate molecular endotypes and treatment-response patterns may exist (Rockel et al., 2025). However, these groups should remain labeled as candidate endotypes until their biological coherence, reproducibility and longitudinal or treatment relevance are independently demonstrated. A statistical cluster should not be termed an endotype solely because it separates participants mathematically. The term endotype should be reserved for a reproducible subgroup with a distinct underlying biological or mechanistic pathway and demonstrated longitudinal or treatment relevance.

## 6 Diagnosis, prediction and patient outcomes

Diagnosis and prediction are among the KOA AI applications closest to clinical translation, but they have different evaluation logic and validation standards. Diagnostic models classify current disease status. Prediction models estimate future event risk and should be evaluated in terms of discrimination, calibration and clinical net benefit. Clinical prediction models should therefore be designed around specific decisions, because early screening, severity grading, structural progression prediction, pain and function prediction, replacement-risk prediction, and postoperative recovery monitoring involve different populations, time windows and misclassification costs (Table 4). Treating all of these tasks as generic KOA prediction weakens model interpretability and hinders integration into care pathways.

### 6.1 Early recognition and assisted image reading

The goal of screening and early recognition is to identify individuals who may benefit from further imaging or clinical assessment. Digital radiographic radiomics nomograms (Sun H. et al., 2026), multimodal radiographic models (Maʼaitah et al., 2025; Sun K. et al., 2026) and Swin Transformer-based radiographic models (Park et al., 2026; Sudha and Rajiv Kannan, 2026) have improved automated grading performance. However, radiographs mainly capture osteophytes, joint-space narrowing and lower-limb alignment, and are less sensitive to cartilage, menisci, synovium and early load abnormalities.

MRI-based AI scoring and OAI-derived MRI scoring systems can add information on cartilage, bone marrow lesions, effusion-synovitis and soft-tissue changes (Roy et al., 2025). Screening models should therefore prioritize sensitivity, negative predictive value, referral thresholds and uncertainty sources, rather than reporting overall accuracy alone.

### 6.2 Progression prediction and patient outcomes

The first task in KOA progression prediction is to define the outcome. A random-forest model developed in OAI and externally validated in MOST by Lee et al. showed that baseline KL grade, contralateral knee OA, lateral joint-space narrowing, body mass index (BMI), medial joint-space narrowing and total Western Ontario and McMaster Universities Osteoarthritis Index (WOMAC) score predicted radiographic progression over four to 5 years (Lee et al., 2025). Wang et al. developed the LBTRBC-M model by integrating 2-year longitudinal DESS MRI radiomics, biochemical biomarkers and clinical variables. In the combined FNIH and OAI test set, the model predicted four end points: joint-space narrowing with pain progression, joint-space narrowing alone, pain progression alone and non-progression. AUCs were 0.880, 0.913, 0.886 and 0.909, respectively, and overall accuracy was 70.1%, indicating that separately modeling structural and symptomatic end points can improve interpretability (Wang et al., 2025). Panfilov et al. further showed in a multimodal Transformer study that structural MRI alone can stably predict progression over two to 8 years, with AUCs of 0.70–0.76. For 1-year short-term progression, fusion of radiography, DESS and T2 mapping contributed more information (Panfilov et al., 2025). The model was more accurate in patients with prior knee injury or surgery, and less accurate on average in those without prior injury.

Structural progression models should avoid treating readily available imaging surrogates as clinical end points. Joint-space narrowing, cartilage-thickness loss and MRI-visible lesion change are meaningful for mechanistic research. Patients, however, are more concerned with pain relief, preserved mobility, work and life impact, and whether surgery will be needed. A prediction model has limited clinical utility unless it explains how its output would alter follow-up interval, imaging frequency, rehabilitation intensity or treatment escalation.

### 6.3 Replacement decisions and recovery trajectories

Replacement decisions and postoperative outcome prediction are relatively mature translational scenarios. Systematic reviews of machine learning for identifying TKA candidates suggest that AI may support candidate identification and referral prioritization for surgical evaluation, but prediction targets should distinguish three situations: imaging suggests possible replacement, the patient is willing to undergo replacement, and surgery is likely to provide meaningful functional benefit (Tian et al., 2025). Perioperative applications have produced more actionable results. In 42 TKA plans, the AIJOINT system reduced CT segmentation time from 128.88 ± 17.31 min to 3.74 ± 0.82 min, and reduced patient-specific instrumentation design time from 159.52 ± 17.14 min to 35.10 ± 3.98 min. Femoral and tibial component-size prediction accuracy was 92.9%, significantly higher than with conventional planning (Li et al., 2023). Prediction of 1-year Forgotten Joint after robotic-assisted TKA (Zhang et al., 2025), AI-based three-dimensional preoperative planning (Lan et al., 2024) and tools for identifying partial knee arthroplasty candidates (York et al., 2024) extend model outputs to implant size selection, axial alignment design, replacement-type decisions and communication about postoperative satisfaction risk. AI should therefore be positioned as a tool for shared decision-making and surgical planning, not as an automated surgical-indication system.

Pain and function should be treated as independent and important prediction end points. Multidimensional plantar-pressure features, interpretable models for fear of falling (Yin et al., 2026), and preoperative sensor-derived Timed Up and Go subtasks and gait parameters (Xie et al., 2024) all indicate that functional status can be quantified using continuous movement data rather than relying solely on imaging severity. Machine learning studies of structural lesions associated with pain further show that effusion-synovitis and cartilage loss are frequent structural abnormalities linked to pain severity, but model AUC remains limited. Pain prediction should therefore incorporate inflammation, structural damage, dynamic loading, psychological factors and activity avoidance (Bacon et al., 2022; Zhao et al., 2024). If AI is to benefit patients, patient-reported outcomes, gait and functional performance and structural phenotypes should be treated jointly as prediction end points. Imaging grade should not be the sole clinical outcome.

## 7 Treatment, rehabilitation and perioperative management

Treatment and rehabilitation are the key settings in which the clinical value of KOA AI must be tested. AI applications in KOA management should move beyond risk prediction toward informing treatment selection and adaptation. Conservative treatment, load-targeted rehabilitation, traditional or integrative rehabilitation, preoperative planning and postoperative follow-up form a continuous management chain (Table 5). If a model output cannot be mapped to a concrete clinical action, its value remains limited to offline performance.

### 7.1 Individualized allocation of conservative treatment

Individualized conservative treatment needs to consider mechanical load, metabolic status and patient adherence simultaneously. Integrative rehabilitation emphasizes exercise, nutrition, biomechanics and physical therapist guidance (Liu et al., 2025). The study by Alkhatatbeh et al. showed that routine clinical variables, such as age and pain severity, outperformed DESS-MRI radiomics in predicting response to hyaluronic acid injection. This result indicates that advanced AI methods must prove their incremental value over variable sets that clinicians can easily obtain (Alkhatatbeh et al., 2026).

Treatment-oriented AI should translate risk prediction into executable treatment suggestions. For patients with obesity and inflammatory pain features, weight management, pain management and sustained exercise may be suitable entry points. For patients with medial compartment overload, gait training, bracing or footwear strategies may be more relevant. For patients with muscle-quality decline, strengthening and nutritional support may be more important (Cowman et al., 2026). Models should help clinicians choose and explain these pathways, rather than simply providing a risk percentage.

### 7.2 Load-targeted rehabilitation

Load-targeted rehabilitation is the most direct application of AI combined with biomechanics. The response of KAM differs substantially among individuals, and uniform gait instructions may be ineffective or may even create adverse load effects in some patients. Models should therefore output individualized adjustment direction and magnitude, rather than a binary label such as internal or external rotation (Baghi et al., 2025a; Plavoukou et al., 2025). Pressure-sensing insoles, IMU-EMG fusion models and real-time KAM feedback are moving estimation of KAM and MJCF toward clinical and home settings (Snyder et al., 2025b). Internet-based rehabilitation, robot-assisted rehabilitation and real-time rotation-sensing systems can improve training dose, adherence and functional feedback, but these technologies should be linked to explicit load targets and patient-reported outcomes (Xie, 2021; Luo et al., 2022; Cai et al., 2023). These digital and robotic technologies provide implementation context but should not be interpreted as direct evidence of AI efficacy unless AI performs a substantive analytical or adaptive function.

### 7.3 Toward perioperative closed-loop management

Perioperative AI applications are beginning to provide components of a potential closed-loop management pathway spanning preoperative planning, surgical support and postoperative monitoring. AI can support preoperative planning for TKA and UKA, component-size prediction, alignment strategy selection, identification of partial knee arthroplasty candidates, and execution optimization during robotic or navigation-assisted surgery (Yang et al., 2023; Longo et al., 2024; Yang et al., 2025). Li et al. showed that the AIJOINT system predicted implant size significantly more accurately than conventional methods (Li et al., 2023). Postoperatively, Timed Up and Go subtask segmentation (Hsieh et al., 2020), wearable-sensor monitoring (Bloomfield et al., 2019), robot-assisted rehabilitation (Scaturro et al., 2023) and models based on early functional-improvement groups (Koo and Hwang, 2020) can identify patients whose recovery trajectory deviates from expectation, indicating a need for intensified rehabilitation or reassessment. Compared with single postoperative scores, continuous movement monitoring may detect early recovery stagnation sooner, but sensor placement, follow-up time points and abnormal-alert thresholds must be standardized (He et al., 2023). Wearable monitoring and robotic rehabilitation should likewise be interpreted as digital implementation context unless an AI component performs a substantive analytical, predictive, or adaptive function.

## 8 Discussion: evidence requirements for clinical translation

Clinical translation requires models to enter real clinical workflows and to produce measurable benefits for clinicians, patients and health systems. In KOA, translational value may include shorter reading time, more precise risk stratification, fewer unnecessary tests, better rehabilitation-resource allocation, improved preoperative counseling and shared decision-making, or greater patient engagement. A single performance metric cannot cover these multidimensional goals.

### 8.1 External validation and reporting standards

Before KOA AI enters clinical practice, the field must move from internal performance reports to reusable evidence systems. External validation should cover different imaging devices, scanning sequences, patient populations, disease stages and clinical workflows. Beyond AUC and accuracy, model evaluation should report calibration, decision-curve analysis, subgroup performance and failure-case analysis. Large cohort resources such as IMI-APPROACH (van Helvoort et al., 2020), translational reviews of orthopedic AI (Pontoh et al., 2026), and reviews of AI in KOA diagnosis and arthroplasty outcome prediction (Lee et al., 2022; Joseph et al., 2025) collectively indicate that standardized, multicenter and longitudinal validation is necessary before clinical implementation.

The current evidence base should therefore be interpreted cautiously. Much of the reported performance arises from retrospective analyses, single-center samples, public cohorts, or internal cross-validation. Repeated analyses of the Osteoarthritis Initiative and Multicenter Osteoarthritis Study do not necessarily constitute independent replication when participants, imaging pipelines, labels, or preprocessing strategies overlap. High discrimination in an internally validated dataset should not be interpreted as evidence of transportability, clinical effectiveness, or patient benefit.

### 8.2 Sources of heterogeneity and limits of cross-study comparison

Substantial methodological and clinical heterogeneity limited direct comparison across the reviewed studies. Study populations ranged from individuals at risk of KOA or with early disease to patients undergoing arthroplasty, and several publications repeatedly analyzed overlapping OAI, MOST or FNIH datasets. Apparent replication across such studies therefore did not always represent independent validation. Data acquisition also varied in radiographic positioning, MRI sequence and scanner characteristics, gait task, sensor type and placement, calibration procedures, and monitoring duration.

Additional heterogeneity arose from model architecture, feature selection and biomechanical assumptions. Finite-element and musculoskeletal models differed in geometry, material properties, boundary conditions and load estimation, whereas imaging and wearable studies used different preprocessing and feature-extraction pipelines. Outcome definitions included KL grade, joint-space narrowing, cartilage loss, pain progression, functional decline, treatment response and arthroplasty-related outcomes over different follow-up periods. Validation ranged from image- or knee-level random splitting to participant-level cross-validation and independent external cohorts. Performance was also reported using non-equivalent metrics, including Dice coefficients, AUC, accuracy, sensitivity, correlation and estimation error. Consequently, differences in reported performance may reflect study design, data partitioning, outcome prevalence and measurement protocols rather than true superiority of one AI method over another.

### 8.3 Task-specific validation metrics

The same model faces different validation requirements in different applications. Imaging-segmentation models should report Dice coefficient, surface distance, external validation and error propagation. For example, conventional MRI cartilage segmentation can maintain a Dice coefficient greater than 0.80 in an external validation set, but performance still differs across cartilage compartments (Yao et al., 2026). Classification models should report calibration and performance under class imbalance. Clinical prediction models should report decision-curve analysis and misclassification costs. Remote-monitoring systems should report device adherence, missing-data handling and abnormal-alert processes. Without this information, even models with high AUCs may not gain clinician trust. Without comparison against simple clinical models, the incremental value of complex AI models remains difficult to judge (Osis et al., 2017).

### 8.4 Interpretability oriented toward clinical action

Interpretability should serve clinical action. Explainable AI studies using contrastive learning (Gao et al., 2025) and fuzzy feature selection (Kokkotis et al., 2022) show that models can highlight imaging regions or features associated with KL grade or KOA diagnosis. Heat maps and feature-importance scores do not, by themselves, establish clinical value. A higher level of explanation should answer action-oriented questions: does the risk mainly arise from structural lesions, abnormal dynamic loading, muscle-quality decline or pain behavior? Can these factors be modified by weight loss, bracing, gait training, further imaging or surgical planning?

### 8.5 Regulatory, economic and implementation requirements

Ethical governance and implementation research are equally important. Successful translation of AI-enabled KOA systems requires coordinated consideration of regulatory compliance, economic value, workforce competency, patient acceptance and health equity.

#### 8.5.1 Regulatory pathways

Regulatory requirements should be considered early in the development of AI-enabled KOA systems rather than after model validation. In the United States, software intended to diagnose KOA, predict progression or support treatment planning may be regulated as a device software function or Software as a Medical Device, whereas certain clinical decision-support functions may fall outside the medical-device definition when the statutory non-device criteria are met. Regulated software follows classification-dependent premarket pathways, and planned algorithm modifications may be addressed through a predetermined change control plan when accepted as part of the marketing submission (U.S. Food and Drug Administration, 2025; U.S. Food and Drug Administration, 2026).

In the European Union, AI-enabled medical-device software must first satisfy the requirements of the Medical Device Regulation, including risk classification, clinical evaluation, quality management, conformity assessment and post-market surveillance. An AI system that is itself a medical device, or that functions as a safety component of a medical device requiring third-party conformity assessment, may additionally be classified as a high-risk AI system under the Artificial Intelligence Act. Relevant requirements include risk management, data governance, technical documentation, human oversight, accuracy, cybersecurity and post-market monitoring (European Parliament and Council of the European Union, 2017; European Parliament and Council of the European Union, 2024).

In China, the regulatory pathway is similarly determined by the intended medical purpose and product risk. AI-based medical software should be classified according to the National Medical Products Administration principles and follow the applicable class-based filing or registration process. Class I devices are generally subject to filing, whereas Class II and Class III devices require registration and technical review by the corresponding regulatory authority. Developers should therefore define the intended users, target population, clinical role, input data, output interpretation, algorithm-update policy and post-market monitoring strategy before registration (National Medical Products Administration, 2021a; National Medical Products Administration, 2021b).

Across jurisdictions, regulatory authorization should not be treated as permanent evidence of clinical effectiveness. KOA models may be affected by changes in imaging devices, MRI sequences, patient populations, wearable-sensor configurations and clinical workflows. Regulatory planning should therefore be linked to version control, cybersecurity, local performance verification, model-drift monitoring and predefined procedures for algorithm modification or withdrawal (World Health Organization, 2023).

#### 8.5.2 Cost-effectiveness and budget impact

Regulatory authorization and predictive performance do not establish affordability or health-system value. Economic evaluation should compare AI-assisted care with current clinical practice and account for software acquisition, licensing, hardware, data storage, interoperability, cybersecurity, maintenance, model updating, staff training and additional clinical review. Potential savings may arise from shorter image-reading time, more targeted imaging, improved rehabilitation-resource allocation, earlier identification of poor recovery and avoidance of ineffective interventions. However, these benefits should be demonstrated using prospective resource-use data rather than inferred from model accuracy alone.

Budget-impact analysis should estimate the eligible population, adoption rate, implementation costs and short-term financial consequences for the relevant institution or health system. Technologies requiring substantial investment or major workflow changes should also undergo cost-effectiveness or cost–utility analysis, with explicit comparison against existing clinical models and care pathways. Sensitivity analyses should consider uncertainty in uptake, device replacement, staff time, algorithm updating and downstream treatment use (National Institute for Health and Care Excellence, 2022). If AI increases clinician review time, produces excessive false alerts or requires substantial additional workflow resources, it may fail to provide clinical or economic value despite strong offline performance.

#### 8.5.3 Workforce training and competency

Clinical deployment also requires competency-based workforce preparation. Orthopedic surgeons, radiologists, rehabilitation professionals, nurses and data-support personnel should understand the intended use of the model, required data quality, uncertainty of the output, common failure modes and populations in which performance has not been established. Training should also specify when an AI recommendation may be overridden, when additional clinical assessment is required and how suspected errors, performance drift or safety incidents should be reported.

Institutions should define responsibility for reviewing AI outputs, resolving conflicts between model recommendations and clinical judgment, maintaining software versions and monitoring post-deployment performance. Competency assessment should extend beyond basic software operation to include critical interpretation and communication of uncertainty. AI should support rather than displace professional accountability, and clinical teams should retain the authority to reject recommendations that are inconsistent with the patient’s condition or preferences (World Health Organization, 2021; National Institute for Health and Care Excellence, 2022; World Health Organization, 2023).

#### 8.5.4 Patient acceptance and equity

Wearable devices and digital platforms continuously generate data on physical activity, pain, sleep quality and rehabilitation adherence. The scope of informed consent, secondary data use, alert thresholds, false-alarm management and responsibility for responding to abnormal results should therefore be clearly defined. Participatory research protocols further suggest that clinical teams and patients should jointly define acceptable data-sharing practices, communication methods and clinical workflows before model deployment (Farrow et al., 2022; Lindsay and Astephen Wilson, 2026; Wang et al., 2026).

Patient acceptance should be evaluated as a clinical implementation outcome rather than assumed from technical performance. Patients should receive understandable information about the purpose of the AI system, the data being collected, the role of human review, the consequences of false-positive or false-negative results and the available alternatives. Participatory design involving patients and clinicians may help identify acceptable data-sharing practices, alert thresholds, communication methods and consent procedures.

Equity assessment should examine whether model performance and access differ by age, sex, ethnicity, socioeconomic status, language, digital literacy, disease severity and geographic setting. Systems that depend on high-cost MRI, repeated wearable monitoring, stable internet access or technically demanding self-management may exclude rural, older or socioeconomically disadvantaged patients. Studies should therefore report subgroup performance, missing-data patterns, device adherence and access barriers, and should retain a clinically appropriate non-digital pathway for patients who cannot or do not wish to use AI-enabled services (World Health Organization, 2021; World Health Organization, 2023).

### 8.6 Future directions

Repeated reuse of large public cohorts such as OAI and MOST has accelerated model development but may also create a form of dataset monoculture. Apparent replication across publications does not necessarily constitute independent external validation when cohorts, participants or acquisition pipelines overlap. A recent global bibliometric analysis of 1,087 publications from 2010 to 2025 showed that research on AI-based KOA progression prediction has shifted from automated radiographic grading toward multimodal and interpretable models integrating imaging, clinical variables, patient-reported outcomes and pain trajectories. This trend supports the multimodal risk-stratification framework proposed in this review, while also highlighting persistent gaps in external validation, cohort diversity and clinical implementation (Özdemir and Topsakal, 2026).

Priorities for the next stage of KOA AI research include building reusable multicenter data standards; embedding biomechanical constraints into model design; defining model-output targets according to clinical decision needs; conducting prospective clinical-impact evaluations; and integrating comprehensive rehabilitation, perioperative management and patient-reported outcomes into a unified follow-up framework. More importantly, future studies should prespecify the clinical action triggered by model output, such as changes in follow-up interval, rehabilitation prescription, brace selection, imaging examination, surgical consultation or shared decision-making. AI is likely to move from an assisted recognition tool to a precision-management system for KOA only when model outputs are linked to these actions and their benefits.

### 8.7 Limitations of this review

This review has several limitations. First, it was designed as a structured narrative review rather than a systematic review or meta-analysis. Although structured database searches, operational eligibility criteria, record counts, and a PRISMA-style flow diagram were used to improve transparency, representative studies were selected according to their relevance to the conceptual and translational framework, and the review should not be interpreted as an exhaustive evidence synthesis.

Second, dual independent screening was not performed, and inter-rater agreement was not calculated. Subjective decisions during study selection and narrative weighting may therefore have introduced selection or interpretation bias.

Third, formal study-level risk-of-bias tools were not applied. The included literature encompassed heterogeneous diagnostic, prognostic, observational, engineering, computational, interventional, and implementation designs, for which no single assessment instrument was appropriate. A structured domain-based critical appraisal was used instead, but this approach does not replace formal risk-of-bias assessment or certainty-of-evidence grading.

Fourth, repeated use of large public cohorts, particularly the Osteoarthritis Initiative and Multicenter Osteoarthritis Study, may limit the independence of apparently replicated findings. Imaging protocols, sensor configurations, biomechanical assumptions, outcome definitions, follow-up periods, and validation strategies also differed substantially across studies, limiting direct comparison.

Accordingly, the conclusions of this review should be interpreted as a critical narrative synthesis of technical feasibility, predictive evidence, and translational boundaries rather than as a quantitative estimate of effect, a formal ranking of model quality, or definitive evidence of clinical efficacy.

## 9 Conclusion

KOA onset, progression and treatment response reflect the combined effects of joint structural degeneration, dynamic mechanical loading, muscle function, pain behavior and systemic metabolic status. AI provides new measurement and integration tools for this complex system, allowing research to move beyond traditional radiographic grading and single-visit assessment toward multimodal analysis of MRI tissue phenotypes, gait mechanical load, wearable activity trajectories, finite element simulation and patient-reported outcomes. The available evidence most consistently supports the technical feasibility of AI-assisted imaging segmentation, severity recognition, and selected biomechanical estimation tasks. Evidence for progression prediction, rehabilitation adaptation, treatment allocation, perioperative decision support, and improved patient outcomes is less mature and is frequently limited by retrospective design, internal validation, heterogeneous outcome definitions, and insufficient comparison with simpler clinical approaches. However, different study designs support different types of inference. Cross-sectional models primarily identify associations and candidate risk markers. Longitudinal studies provide evidence for prediction and risk stratification, whereas intervention and implementation studies offer more direct evidence regarding clinical utility. These categories should not be interpreted as a universal hierarchy of methodological quality, because study validity also depends on design, sample size, external validation and risk of bias. Future AI-enabled knee osteoarthritis systems should therefore be evaluated through independent external validation, calibration, prospective clinical-impact studies, and patient-centered outcomes before they are considered suitable for routine clinical decision-making. The present review identifies promising applications and evidence gaps but does not establish the clinical efficacy of AI-based interventions.

## References

[B1] Ali Z. Moon J. Gillani S. Afzal S. Maqsood M. Rho S. (2025). Vision-based approach to knee osteoarthritis and Parkinson's disease detection utilizing human gait patterns. PeerJ Comput. Sci. 11, e2857. 10.7717/peerj-cs.2857 PMC12192726 40567719

[B2] Alkhatatbeh T. Alkhatatbeh A. Liao Y. Zhang Z. Fang H. Chen W. (2026). Clinical features outperform MRI radiomics for predicting intra-articular hyaluronic acid response in knee osteoarthritis. Ann. N. Y. Acad. Sci. 1559 (1), e70288. 10.1111/nyas.70288 42179107

[B3] Aragón-Basanta E. Page A. Ayala G. Viosca-Herrero E. Alabajos-Cea A. Herrero-Manley L. (2025). Functional data analysis of ground reaction forces combined with clinical measures for early knee osteoarthritis detection. Sci. Rep. 15 (1), 40514. 10.1038/s41598-025-24411-y PMC12627083 41254098

[B4] Bacon K. L. Felson D. T. Jafarzadeh S. R. Kolachalama V. B. Hausdorff J. M. Gazit E. (2022). Relation of gait measures with mild unilateral knee pain during walking using machine learning. Sci. Rep. 12 (1), 22200. 10.1038/s41598-022-21142-2 PMC9789148 36564397

[B5] Bacon K. L. Felson D. T. Jafarzadeh S. R. Kolachalama V. B. Hausdorff J. M. Gazit E. (2024). Gait alterations and association with worsening knee pain and physical function: a machine learning approach with wearable sensors in the multicenter osteoarthritis study. Arthritis Care Res. Hob. 76 (7), 984–992. 10.1002/acr.25327 PMC11209802 38523250

[B6] Bacon K. L. Felson D. T. Jafarzadeh S. R. Hausdorff J. M. Gazit E. Guermazi A. (2026). Association of wearable sensor-derived gait measures with cartilage damage over two years among adults with or at risk of knee osteoarthritis. Arthritis Care Res. Hob. 78 (9), 1229–1238. 10.1002/acr.80018 PMC13098771 41685951

[B7] Baghi R. Yin W. Badhyal S. Ramadan A. Oppizzi G. Li Z. (2025a). Determining the individual relationship between the step width and peak knee adduction moment during stepping in medial knee osteoarthritis. Clin. Biomech. (Bristol) 128, 106619. 10.1016/j.clinbiomech.2025.106619 PMC12368841 40737736

[B8] Baghi R. Yin W. Ramadan A. Badhyal S. Oppizzi G. Xu D. (2025b). Determining individualized foot progression angle for reduction of knee medial compartment loading during stepping. Med. Sci. Sports Exerc 57 (1), 33–43. 10.1249/mss.0000000000003531 PMC12411098 39186734

[B9] Bercaw J. R. Bradley P. X. Otap C. C. Heckelman L. N. Tamayo K. S. Owusu-Akyaw K. A. (2025). A deep learning-based approach for measuring patellar cartilage deformations from knee MR images. J. Biomech. 192, 112930. 10.1016/j.jbiomech.2025.112930 PMC12781745 40915052

[B10] Bharanidivya M. Dhanalakshmi S. (2026). TinyML-enabled wearable system for early detection of knee osteoarthritis using ensemble gait classification. Comput. Biol. Med. 200, 111345. 10.1016/j.compbiomed.2025.111345 41353887

[B11] Binvignat M. Sellam J. Berenbaum F. Felson D. T. (2024). The role of obesity and adipose tissue dysfunction in osteoarthritis pain. Nat. Rev. Rheumatol. 20 (9), 565–584. 10.1038/s41584-024-01143-3 39112603

[B12] Bliddal H. Bays H. Czernichow S. Uddén Hemmingsson J. Hjelmesæth J. Hoffmann Morville T. (2024). Once-weekly semaglutide in persons with obesity and knee osteoarthritis. N. Engl. J. Med. 391 (17), 1573–1583. 10.1056/NEJMoa2403664 39476339

[B13] Bloomfield R. A. Williams H. A. Broberg J. S. Lanting B. A. McIsaac K. A. Teeter M. G. (2019). Machine learning groups patients by early functional improvement likelihood based on wearable sensor instrumented preoperative timed-up-and-go tests. J. Arthroplasty 34 (10), 2267–2271. 10.1016/j.arth.2019.05.061 31255408

[B14] Cai L. Liu Y. Wei Z. Liang H. Liu Y. Cui M. (2023). Robot-assisted rehabilitation training improves knee function and daily activity ability in older adults following total knee arthroplasty. Res. Nurs. Health 46 (2), 203–209. 10.1002/nur.22290 36504201

[B15] Chavoshi M. Trivedi H. Newsome J. Mansuri A. Li F. Dapamede T. (2026). Comparative evaluation of deep learning and foundation model embeddings for osteoarthritis feature classification in knee radiographs. J. Imaging Inf. Med. 39 (3), 2136–2149. 10.1007/s10278-025-01636-x PMC13230456 40897834

[B16] Consolini J. Koch K. M. Burge A. J. Xia E. Majumdar S. Gold G. E. (2025). Exploratory analysis of infrapatellar fat pad MRI-based radiomics for detection of knee structure abnormalities in collegiate basketball players and swimmers. Osteoarthr. Imaging 5 (4), 100379. 10.1016/j.ostima.2025.100379 PMC13228674 42239636

[B17] Costello K. E. Felson D. T. Jafarzadeh S. R. Guermazi A. Roemer F. W. Segal N. A. (2023). Gait, physical activity and tibiofemoral cartilage damage: a longitudinal machine learning analysis in the multicenter osteoarthritis study. Br. J. Sports Med. 57 (16), 1018–1024. 10.1136/bjsports-2022-106142 PMC10423491 36868795

[B18] Cowman A. W. Tang J. N. Deng J. Abu-Halawa A. Jin H. Liu C. Z. (2026). Current and emerging therapies for knee osteoarthritis: from conventional approaches to machine learning and stem cell innovations. Regen. Ther. 32, 101106. 10.1016/j.reth.2026.101106 PMC13133973 42077270

[B19] Dang Q. Hong Z. Zeng D. Cao P. Li S. Cen H. (2026). MRI-based radiomics to determine the role of thigh muscle quality in the development of knee osteoarthritis: a discovery and validation study. J. Adv. Res. 79, 819–831. 10.1016/j.jare.2025.04.013 PMC12766218 40222453

[B20] Diamond L. E. Grant T. Uhlrich S. D. (2024). Osteoarthritis year in review 2023: biomechanics. Osteoarthr. Cartil. 32 (2), 138–147. 10.1016/j.joca.2023.11.015 38043858

[B21] Ebrahimkhani S. Jaward M. H. Cicuttini F. M. Dharmaratne A. Wang Y. de Herrera A. G. S. (2020). A review on segmentation of knee articular cartilage: from conventional methods towards deep learning. Artif. Intell. Med. 106, 101851. 10.1016/j.artmed.2020.101851 32593389

[B22] European Parliament and Council of the European Union (2017). Regulation (EU) 2017/745 of the European parliament and of the council of 5 April 2017 on medical devices. J. Eur. Union L117, 1–175. Available online at: https://eur-lex.europa.eu/eli/reg/2017/745/oj/eng.

[B23] European Parliament and Council of the European Union (2024). Regulation (EU) 2024/1689 laying down harmonised rules on artificial intelligence. J. Eur. Union 2024/1689. Available online at: https://eur-lex.europa.eu/eli/reg/2024/1689/oj/eng.

[B24] Farrow L. Ashcroft G. P. Zhong M. Anderson L. (2022). Using artificial intelligence to revolutionise the patient care pathway in hip and knee arthroplasty (ARCHERY): protocol for the development of a clinical prediction model. JMIR Res. Protoc. 11 (5), e37092. 10.2196/37092 PMC9133991 35544289

[B25] Feng Z. Zhu D. Wu T. Li H. Han T. (2025). Self-aware fusion IMU-EMG attention dependence for knee adduction moment estimation during walking. IEEE J. Biomed. Health Inf. 29 (9), 6496–6509. 10.1109/jbhi.2025.3564981 40293894

[B26] Fox S. Ciliberti F. K. Jónsson H. Jr. Gargiulo P. Recenti M. (2026). Impact of automated and manual segmentation errors on knee osteoarthritis classification using MRI-registered data on CT scans. Comput. Med. Imaging Graph 131, 102767. 10.1016/j.compmedimag.2026.102767 41985396

[B27] Gao F. Littlefield N. Myers N. Yates A. J. Weiss K. R. Plate J. F. (2025). Explainable contrastive learning for KL grading classification in knee osteoarthritis. Annu. Int. Conf. IEEE Eng. Med. Biol. Soc. 2025, 1–7. 10.1109/embc58623.2025.11252884 41336856

[B28] GBD 2021 Osteoarthritis Collaborators (2023). Global, regional, and national burden of osteoarthritis, 1990-2020 and projections to 2050: a systematic analysis for the global burden of disease study 2021. Lancet Rheumatol. 5 (9), e508–e522. 10.1016/s2665-9913(23)00163-7 PMC10477960 37675071

[B29] Guan S. Mei Y. (2026). Biomechanics and obesity in osteoarthritis: from mechanism to precision medicine. Int. J. Rheum. Dis. 29 (1), e70539. 10.1111/1756-185x.70539 41566632

[B30] Guo H. Kang Z. Bai X. Tian X. Jin F. (2025). Characteristics and advantages in finite element analysis techniques in knee biomechanics. Chin. J. Tissue Eng. Res. 29 (15), 3253–3261.

[B31] Havashinezhadian S. Chiasson-Poirier L. Sylvestre J. Turcot K. (2023). Inertial sensor location for ground reaction force and gait event detection using reservoir computing in gait. Int. J. Environ. Res. Public Health 20 (4), 3120. 10.3390/ijerph20043120 PMC9962509 36833815

[B32] He R. Xiong R. Sun M. L. Yang J. J. Chen H. Yang P. F. (2023). Study on the correlation between early three-dimensional gait analysis and clinical efficacy after robot-assisted total knee arthroplasty. Chin. J. Traumatol. 26 (2), 83–93. 10.1016/j.cjtee.2022.05.003 PMC10071330 35798637

[B33] Hodgkinson T. Kelly D. C. Curtin C. M. O'Brien F. J. (2022). Mechanosignalling in cartilage: an emerging target for the treatment of osteoarthritis. Nat. Rev. Rheumatol. 18 (2), 67–84. 10.1038/s41584-021-00724-w 34934171

[B34] Hoyer G. Gao K. T. Gassert F. G. Luitjens J. Jiang F. Majumdar S. (2025). Foundations of a knee joint digital twin from qMRI biomarkers for osteoarthritis and knee replacement. NPJ Digit. Med. 8 (1), 118. 10.1038/s41746-025-01507-3 PMC11845592 39984725

[B35] Hsieh C. Y. Huang H. Y. Liu K. C. Chen K. H. Hsu S. J. Chan C. T. (2020). Subtask segmentation of timed up and Go test for mobility assessment of perioperative total knee arthroplasty. Sensors (Basel) 20 (21), 6302. 10.3390/s20216302 33167444 PMC7663910

[B36] Hwang U. J. Chung K. S. Ha S. M. (2025a). Clustering and classification of early knee osteoarthritis using machine-learning analysis of step-up and down test kinematics in recreational table tennis players. PeerJ 13, e19471. 10.7717/peerj.19471 PMC12124290 40452943

[B37] Hwang U. J. Chung K. S. Ha S. M. (2025b). Machine learning for clustering and classification of early knee osteoarthritis using single-leg standing kinematics. Digit. Health 11, 20552076251326226. 10.1177/20552076251326226 PMC11907616 40093697

[B38] Jarraya M. Guermazi A. Roemer F. W. (2024). Osteoarthritis year in review 2023: imaging. Osteoarthr. Cartil. 32 (1), 18–27. 10.1016/j.joca.2023.10.005 37879600

[B39] Jayakumar P. Rathouz P. J. Lin E. Trutner Z. Uhler L. M. Andrawis J. (2025). Shared decision making using digital twins in knee osteoarthritis care: a randomized clinical trial of an AI-enabled decision aid versus education alone on decision quality, physical function, and user experience. EClinicalMedicine 89, 103545. 10.1016/j.eclinm.2025.103545 PMC12528923 41112505

[B40] Jiang S. Li H. Zhang L. Mu W. Zhang Y. Chen T. (2025). Generic diagramming platform (GDP): a comprehensive database of high-quality biomedical graphics. Nucleic Acids Res. 53 (D1), D1670–d1676. 10.1093/nar/gkae973 PMC11701665 39470721

[B41] Joseph G. B. McCulloch C. E. Nevitt M. C. Lane N. E. Majumdar S. Link T. M. (2025). Machine learning models for clinical and structural knee osteoarthritis prediction: recent advancements and future directions. Osteoarthr. Cartil. Open 7 (3), 100654. 10.1016/j.ocarto.2025.100654 PMC12341514 40799630

[B42] Kaya E. Şirzai H. Yavuzer G. Argunsah H. (2025). Classification of knee osteoarthritis severity using markerless motion capture and long short-term memory fully convolutional network. Comput. Biol. Med. 195, 110647. 10.1016/j.compbiomed.2025.110647 40582166

[B43] Kijowski R. Fritz J. Deniz C. M. (2023). Deep learning applications in osteoarthritis imaging. Skelet. Radiol. 52 (11), 2225–2238. 10.1007/s00256-023-04296-6 PMC10409879 36759367

[B44] Kloppenburg M. Namane M. Cicuttini F. (2025). Osteoarthritis. Lancet 405 (10472), 71–85. 10.1016/s0140-6736(24)02322-5 39755397

[B45] Kluge F. Hannink J. Pasluosta C. Klucken J. Gaßner H. Gelse K. (2018). Pre-operative sensor-based gait parameters predict functional outcome after total knee arthroplasty. Gait Posture 66, 194–200. 10.1016/j.gaitpost.2018.08.026 30199778

[B46] Kokkotis C. Ntakolia C. Moustakidis S. Giakas G. Tsaopoulos D. (2022). Explainable machine learning for knee osteoarthritis diagnosis based on a novel fuzzy feature selection methodology. Phys. Eng. Sci. Med. 45 (1), 219–229. 10.1007/s13246-022-01106-6 PMC8802106 35099771

[B47] Koo K. I. Hwang C. H. (2020). Five-day rehabilitation of patients undergoing total knee arthroplasty using an end-effector gait robot as a neuromodulation blending tool for deafferentation, weight offloading and stereotyped movement: interim analysis. PLoS One 15 (12), e0241117. 10.1371/journal.pone.0241117 PMC7743990 33326434

[B48] Kostic A. M. Arbeeva L. Jiang X. Golightly Y. M. Messier S. P. Loeser R. F. (2025). Determining optimal diet/exercise treatment assignment for patients with symptomatic knee osteoarthritis using baseline gait forces. Osteoarthr. Cartil. Open 7 (4), 100691. 10.1016/j.ocarto.2025.100691 PMC12546872 41141647

[B49] Kwon S. B. Ro D. H. Song M. K. Han H. S. Lee M. C. Kim H. C. (2019). Identifying key gait features associated with the radiological grade of knee osteoarthritis. Osteoarthr. Cartil. 27 (12), 1755–1760. 10.1016/j.joca.2019.07.014 31400498

[B50] Lan Q. Li S. Zhang J. Guo H. Yan L. Tang F. (2024). Reliable prediction of implant size and axial alignment in AI-based 3D preoperative planning for total knee arthroplasty. Sci. Rep. 14 (1), 16971. 10.1038/s41598-024-67276-3 PMC11266554 39043748

[B51] Lau S. H. Chan L. C. Jiang T. Zhang J. Meng X. Wang W. (2025). Diffusion model-empowered patella shape analysis predicts knee osteoarthritis outcomes. Osteoarthr. Cartil. Open 7 (4), 100663. 10.1016/j.ocarto.2025.100663 PMC12409384 40919067

[B52] Lee L. S. Chan P. K. Wen C. Fung W. C. Cheung A. Chan V. W. K. (2022). Artificial intelligence in diagnosis of knee osteoarthritis and prediction of arthroplasty outcomes: a review. Arthroplasty 4 (1), 16. 10.1186/s42836-022-00118-7 PMC8897859 35246270

[B53] Lee D. W. Han H. S. Lee M. C. Ro D. H. (2024). Prediction of postoperative gait speed change after bilateral primary total knee arthroplasty in female patients using a machine learning algorithm. Orthop. Traumatol. Surg. Res. 110 (7), 103842. 10.1016/j.otsr.2024.103842 38382881

[B54] Lee H. S. Hwang S. Kim S. H. Joon N. B. Kim H. Hong Y. S. (2024). Automated analysis of knee joint alignment using detailed angular values in long leg radiographs based on deep learning. Sci. Rep. 14 (1), 7226. 10.1038/s41598-024-57887-1 PMC10973521 38538685

[B55] Lee D. W. Han H. S. Ro D. H. Lee Y. S. (2025). Development of the machine learning model that is highly validated and easily applicable to predict radiographic knee osteoarthritis progression. J. Orthop. Res. 43 (1), 128–138. 10.1002/jor.25982 39354808

[B56] Li S. Liu X. Chen X. Xu H. Zhang Y. Qian W. (2023). Development and validation of an artificial intelligence preoperative planning and patient-specific instrumentation system for total knee arthroplasty. Bioeng. (Basel) 10 (12), 1417. 10.3390/bioengineering10121417 PMC10740483 38136008

[B57] Li S. Yao Y. Zhong J. Zhao S. Xiao F. Ong T. M. (2026). ERANet: edge replacement augmentation for semi-supervised meniscus segmentation with prototype consistency alignment and conditional self-training. Neural Netw. 196, 108337. 10.1016/j.neunet.2025.108337 41270438

[B58] Lindsay O. R. Astephen Wilson J. L. (2026). Integrating gait analysis and AI into knee osteoarthritis care: protocol for a 3-Phase participatory qualitative study. JMIR Res. Protoc. 15, e82860. 10.2196/82860 PMC13075632 41973876

[B59] Liu M. Ma P. Yin C. Jiang K. Ju X. (2024). Three-dimensional finite element analysis of the effects of different bone densities on the structures within the knee joint after unicompartmental knee arthroplasty. Chin. J. Tissue Eng. Res. 28 (24), 3801–3806.

[B60] Liu H. Qin L. Liu Y. Meng X. Li C. He M. (2025). Knee osteoarthritis rehabilitation: an integrated framework of exercise, nutrition, biomechanics, and physical therapist guidance-a narrative review. Eur. J. Med. Res. 30 (1), 826. 10.1186/s40001-025-03083-4 PMC12399006 40887656

[B61] Longo U. G. De Salvatore S. Valente F. Villa Corta M. Violante B. Samuelsson K. (2024). Artificial intelligence in total and unicompartmental knee arthroplasty. BMC Musculoskelet. Disord. 25 (1), 571. 10.1186/s12891-024-07516-9 39034416 PMC11265144

[B62] Luo J. Li Y. He M. Wang Z. Li C. Liu D. (2022). Rehabilitation of total knee arthroplasty by integrating conjoint isometric myodynamia and real-time rotation sensing system. Adv. Sci. (Weinh) 9 (8), e2105219. 10.1002/advs.202105219 35038245 PMC8922106

[B63] Maʼaitah M. K. S. Helwan A. Radwan A. Mohammad Salem Manasreh A. Alshareef E. A. (2025). Multimodal model for knee osteoarthritis KL grading from plain radiograph. J. Xray Sci. Technol. 33 (3), 608–620. 10.1177/08953996251314765 40091559

[B64] Mahmoudian A. Haugen I. K. (2026). Rethinking early-stage knee osteoarthritis beyond radiography. Nat. Rev. Rheumatol. 22 (7), 409–410. 10.1038/s41584-026-01389-z 42203902

[B65] Mapinduzi J. Daniels K. Kossi O. Verbrugghe J. Bonnechère B. (2026). Wearable sensors and artificial intelligence for ecological knee osteoarthritis assessment: development and feasibility of a hybrid digital phenotyping framework. Sensors (Basel) 26 (11), 3563. 10.3390/s26113563 42281079 PMC13259533

[B66] McDonald S. M. Felfeliyan B. Hassan A. Küpper J. C. El-Hajj R. Wichuk S. (2025). Evaluating potential for AI automation of quantitative and semi-quantitative MRI scoring in arthritis, especially at the knee: a systematic literature review. Skelet. Radiol. 54 (11), 2339–2349. 10.1007/s00256-025-04922-5 40140064

[B67] Mononen M. E. Turunen M. J. Stenroth L. Saarakkala S. Boesen M. (2024). Biomechanical modeling and imaging for knee osteoarthritis - is there a role for AI? Osteoarthr. Imaging 4 (2), 100182. 10.1016/j.ostima.2024.100182 42238770 PMC13228678

[B68] Moura F. A. Pelegrinelli A. R. M. Catelli D. S. Lamontagne M. da Silva Torres R. (2025). Knee osteoarthritis prediction from gait kinematics: exploring the potential of deep neural networks and transfer learning methods for time series classification. J. Biomech. 190, 112881. 10.1016/j.jbiomech.2025.112881 40749262

[B69] Mu L. Fu J. Li M. Liu H. Dong D. Gong J. (2026). Total knee radiomic feature atlas derived from magnetic resonance imaging segmentation: insights into osteoarthritis incidence predictors. Eur. J. Radiol. 194, 112480. 10.1016/j.ejrad.2025.112480 41187641

[B70] National Institute for Health and Care Excellence (2022). “Evidence standards framework for digital health technologies,” in Corporate Document ECD7 (London: National Institute for Health and Care Excellence).

[B71] National Medical Products Administration (2021a). Announcement on Guidance for the Classification Defining of AI-based Medical Software Products. Beijing: National Medical Products Administration.

[B72] National Medical Products Administration (2021b). Provisions for Medical Device Registration and Filing (SAMR Decree No. 47). Beijing: National Medical Products Administration.

[B73] Nevalainen M. T. Nykänen O. Järvinen J. Kemppainen A. Räsänen L. Casula V. (2026). Clinical feasibility of deep learning contrast synthesis from MR fingerprinting in knee osteoarthritis. J. Magn. Reson Imaging 64 (1), 90–109. 10.1002/jmri.70300 PMC13253990 41882935

[B74] Osis S. T. Kobsar D. Leigh R. J. Macaulay C. A. J. Ferber R. (2017). An expert system feedback tool improves the reliability of clinical gait kinematics for older adults with lower limb osteoarthritis. Gait Posture 58, 261–267. 10.1016/j.gaitpost.2017.08.011 28825998

[B75] Ou D. Ye Y. Pan J. Huang Y. Kuang H. Tang S. (2024). Finite element study of stress distribution in medial UKA under varied lower limb alignment. Sci. Rep. 14 (1), 25397. 10.1038/s41598-024-74145-6 39455644 PMC11511828

[B76] Özdemir E. Topsakal F. E. (2026). AI-based knee osteoarthritis progression prediction: a comprehensive global bibliometric and hotspot evolution analysis (2010-2025). Knee Surg. Relat. Res. 38 (1), 18. 10.1186/s43019-026-00319-3 PMC13130423 42063116

[B77] Panfilov E. Saarakkala S. Nieminen M. T. Tiulpin A. (2025). End-to-End prediction of knee osteoarthritis progression with multimodal transformers. IEEE J. Biomed. Health Inf. 29 (9), 6276–6286. 10.1109/jbhi.2025.3536170 40031337

[B78] Park J. Y. Nam J. H. Abdigapporov S. Kim J. K. Koh Y. G. Cho B. W. (2026). Enhanced magnetic resonance imaging-based knee cartilage segmentation using a Swin-UNet conditional generative adversarial network: development and validation study. JMIR Med. Inf. 14, e86155. 10.2196/86155 PMC12993272 41771536

[B79] Paz A. Lavikainen J. Turunen M. J. García J. J. Korhonen R. K. Mononen M. E. (2024). Knee-loading predictions with neural networks improve finite element modeling classifications of knee osteoarthritis: data from the osteoarthritis initiative. Ann. Biomed. Eng. 52 (9), 2569–2583. 10.1007/s10439-024-03549-2 38842728 PMC11329407

[B80] Plavoukou T. Sotiropoulos S. Taraxidis E. Stasinopoulos D. Georgoudis G. (2025). Sensor technologies and rehabilitation strategies in total knee arthroplasty: current landscape and future directions. Sensors (Basel) 25 (15), 4592. 10.3390/s25154592 40807756 PMC12349383

[B81] Pontoh L. A. P. Hadisoebroto Dilogo I. Fiolin J. Gosal S. Herdiman J. A. Hawali A. A. (2026). Application of artificial intelligence in orthopaedic research: from preclinical to translational. J. Orthop. Surg. (Hong Kong) 34 (1), 10225536261424029. 10.1177/10225536261424029 41680105

[B82] Rani S. Rout A. Soni P. Gupta M. Kumar N. Kumar K. (2026). Review of CNN-based approaches for preprocessing, segmentation and classification of knee osteoarthritis. Diagn. (Basel) 16 (3), 461. 10.3390/diagnostics16030461 PMC12897085 41681779

[B83] Rockel J. S. Sharma D. Espin-Garcia O. Hueniken K. Sandhu A. Pastrello C. (2025). Deep learning-based clustering for endotyping and post-arthroplasty response classification using knee osteoarthritis multiomic data. Ann. Rheum. Dis. 84 (5), 844–855. 10.1016/j.ard.2025.01.012 39948003

[B84] Roy C. Roshan M. Goyal N. Rana P. Ghonge N. P. Jena A. (2025). MRI detection and grading of knee osteoarthritis - a pilot study using an AI technique with a novel imaging-based scoring system. Biomater. Sci. 13 (19), 5475–5494. 10.1039/d5bm00470e 40889152

[B85] Sarmah A. Boruah L. Ito S. Kanagaraj S. (2024). Integrative approach to pedobarography and pelvis-trunk motion for knee osteoarthritis detection and exploration of non-radiographic rehabilitation monitoring. Front. Bioeng. Biotechnol. 12, 1401153. 10.3389/fbioe.2024.1401153 39144481 PMC11321954

[B86] Scaturro D. Vitagliani F. Caracappa D. Tomasello S. Chiaramonte R. Vecchio M. (2023). Rehabilitation approach in robot assisted total knee arthroplasty: an observational study. BMC Musculoskelet. Disord. 24 (1), 140. 10.1186/s12891-023-06230-2 36814210 PMC9945668

[B87] Snyder S. J. Fakhar M. Miller R. H. Bera A. Shim J. K. (2025a). Prediction of knee loads during activities of daily living using custom instrumented insoles and machine learning. J. Biomech. 191, 112921. 10.1016/j.jbiomech.2025.112921 40885049

[B88] Snyder S. J. Lee H. Chu E. Heo Y. J. Miller R. H. Shim J. K. (2025b). Prediction of medial tibiofemoral joint reaction force using custom instrumented insoles and neural networks for walking and running tasks. J. Appl. Biomech. 41 (5), 389–395. 10.1123/jab.2024-0181 40550501

[B89] Sudha K. Rajiv Kannan A. (2026). Enhanced swin transformer with dual attention for knee osteoarthritis severity grading from X-ray images. Sci. Rep. 16 (1), 10617. 10.1038/s41598-026-44174-4 41912656 PMC13039998

[B90] Sun H. Long C. Ma Y. Xing T. Dong S. Guo S. (2026). Radiomics-based nomogram using digital radiography for early diagnosis of knee osteoarthritis. Radiol. Med. 131 (5), 834–844. 10.1007/s11547-026-02189-x 41670886 PMC13201300

[B91] Sun K. Huang Z. Hang M. Lu W. Zhu J. (2026). Knee osteoarthritis screening using multimodal gait signals transformed via Gramian angular field and integrated by a deep learning model. Med. Eng. Phys. 147 (1), 015002. 10.1088/1873-4030/ae1823 41642217

[B92] Suzuki M. Kinoshita T. Kono K. Soufi M. Otake Y. Uemura K. (2025). Varus deformity in medial knee osteoarthritis correlates with fatty degeneration of lower limb muscles: artificial intelligence-based computed tomography analysis. J. Exp. Orthop. 12 (4), e70518. 10.1002/jeo2.70518 PMC12616397 41245706

[B93] Tan J. S. Tippaya S. Binnie T. Davey P. Napier K. Caneiro J. P. (2022). Predicting knee joint kinematics from wearable sensor data in people with knee osteoarthritis and clinical considerations for future machine learning models. Sensors (Basel) 22 (2), 446. 10.3390/s22020446 35062408 PMC8781640

[B94] Tang S. Zhang C. Oo W. M. Fu K. Risberg M. A. Bierma-Zeinstra S. M. (2025). Osteoarthritis. Nat. Rev. Dis. Prim. 11 (1), 10. 10.1038/s41572-025-00594-6 39948092

[B95] Tian C. Chen H. Shao W. Zhang R. Yao X. Shu J. (2025). Accuracy of machine learning in identifying candidates for total knee arthroplasty (TKA) surgery: a systematic review and meta-analysis. Eur. J. Med. Res. 30 (1), 317. 10.1186/s40001-025-02545-z 40264241 PMC12016301

[B96] U.S. Food and Drug Administration (2025). Marketing Submission Recommendations for a Predetermined Change Control Plan for Artificial intelligence-enabled Device Software Functions: Guidance for Industry and Food and Drug Administration Staff. Silver Spring, MD: U.S. Food and Drug Administration.

[B97] U.S. Food and Drug Administration (2026). Clinical Decision Support Software: Guidance for Industry and Food and Drug Administration Staff. Silver Spring, MD: U.S. Food and Drug Administration.

[B98] van Helvoort E. M. van Spil W. E. Jansen M. P. Welsing P. M. J. Kloppenburg M. Loef M. (2020). Cohort profile: the applied public-private research enabling OsteoArthritis clinical headway (IMI-APPROACH) study: a 2-year, European, cohort study to describe, validate and predict phenotypes of osteoarthritis using clinical, imaging and biochemical markers. BMJ Open 10 (7), e035101. 10.1136/bmjopen-2019-035101 32723735 PMC7389775

[B99] Wang S. Chan P. P. K. Lam B. M. F. Chan Z. Y. S. Zhang J. H. W. Wang C. (2021). Sensor-based gait retraining lowers knee adduction moment and improves symptoms in patients with knee osteoarthritis: a randomized controlled trial. Sensors (Basel) 21 (16), 5596. 10.3390/s21165596 34451039 PMC8402273

[B100] Wang T. Liu H. Zhao W. Cao P. Li J. Chen T. (2025). Predicting knee osteoarthritis progression using neural network with longitudinal MRI radiomics, and biochemical biomarkers: a modeling study. PLoS Med. 22 (8), e1004665. 10.1371/journal.pmed.1004665 40839548 PMC12370028

[B101] Wang F. Wang L. Zhong L. Feng J. Wang X. (2026). Artificial intelligence for knee osteoarthritis care and rehabilitation: a systematic review. Pain Manag. Nurs. 27 (1), 76–85. 10.1016/j.pmn.2025.07.013 40849241

[B102] World Health Organization (2021). Ethics and Governance of Artificial Intelligence for Health: WHO Guidance. Geneva: World Health Organization.

[B103] World Health Organization (2023). Regulatory Considerations on Artificial Intelligence for Health. Geneva: World Health Organization.

[B104] Xie S. (2021). A Randomized Controlled Trial of Internet-based Rehabilitation for Patients with Knee Osteoarthritis. Master's thesis. Chengdu: Sichuan University.

[B105] Xie M. Li T. Wei X. (2023). Research progress on finite element modeling and analysis of knee osteoarthritis degeneration. Chin. J. Stereology Image Analysis 28 (3), 279–284. 10.13505/j.1007-1482.2023.28.03.006

[B106] Xie J. Li S. Song Z. Shu L. Zeng Q. Huang G. (2024). Functional monitoring of patients with knee osteoarthritis based on multidimensional wearable plantar pressure features: cross-sectional study. JMIR Aging 7, e58261. 10.2196/58261 39586093 PMC11629041

[B107] Yang Y. Z. Cheng Q. H. Zhang A. R. Qiu Y. Guo H. Z. (2023). Research progress of functional alignment robot assisted knee replacement. Zhongguo Gu Shang 36 (11), 1106–1110. 10.12200/j.issn.1003-0034.2023.11.019 38012884

[B108] Yang J. Ren P. Xin P. Wang Y. Ma Y. Liu W. (2024). Automatic measurement of lower limb alignment in portable devices based on deep learning for knee osteoarthritis. J. Orthop. Surg. Res. 19 (1), 232. 10.1186/s13018-024-04658-3 38594698 PMC11005281

[B109] Yang X. Cheng Q. Zhang F. Fan H. (2025). Early impact of robot assisted total knee arthroplasty on the treatment of varus knee arthritis. China J. Orthop. Traumatol. 38 (4), 343–351. 10.12200/j.issn.1003-0034.20230777 40296594

[B110] Yao L. Liang X. Liu J. Qian B. Xiao Z. Zhu D. (2026). Deep learning-enabled segmentation of knee cartilage in conventional magnetic resonance images: internal and external validation of different models. Knee 58, 104270. 10.1016/j.knee.2025.10.024 41241647

[B111] Yeoh P. S. Q. Lai K. W. Goh S. L. Hasikin K. Hum Y. C. Tee Y. K. (2021). Emergence of deep learning in knee osteoarthritis diagnosis. Comput. Intell. Neurosci. 2021, 4931437. 10.1155/2021/4931437 PMC8598325 34804143

[B112] Yin M. Fang W. Cheng Y. Feng Y. (2026). Personalizing fall fear prevention in knee osteoarthritis: an interpretable prediction framework via IGKSO synchronous optimization and SHAP-based risk stratification. Front. Public Health 14, 1749921. 10.3389/fpubh.2026.1749921 41835436 PMC12979443

[B113] York T. J. Szyszka B. Brivio A. Musbahi O. Barrett D. Cobb J. P. (2024). A radiographic artificial intelligence tool to identify candidates suitable for partial knee arthroplasty. Arch. Orthop. Trauma Surg. 144 (11), 4963–4968. 10.1007/s00402-024-05589-8 39361058 PMC11582309

[B114] Yu A. S. Yang M. Nakamura K. Lartey R. Tosun S. Subhas N. (2026). Multi-label ensemble model for knee joint anatomy and lesion segmentation: segmentation of clinical images with ensembling, morphology, and attention (SCEMA). Magn. Reson Med. 96 (2), 918–927. 10.1002/mrm.70365 41915620 PMC13063831

[B115] Zhang C. Wang Z. Zhang J. Wang Q. Yang D. Zhou Y. (2025). Development and validation of a nomogram for predicting a forgotten joint in patients one year after robotic-assisted total knee arthroplasty. BMC Musculoskelet. Disord. 26 (1), 536. 10.1186/s12891-025-08789-4 40450270 PMC12125914

[B116] Zhang Z. Luo W. Liu C. (2026). Application value of finite element analysis on unicompartmental knee arthroplasty for medial knee compartmental osteoarthritis. Chin. J. Tissue Eng. Res. 30 (9), 2313–2322.

[B117] Zhao Z. Zhao M. Yang T. Li J. Qin C. Wang B. (2024). Identifying significant structural factors associated with knee pain severity in patients with osteoarthritis using machine learning. Sci. Rep. 14 (1), 14705. 10.1038/s41598-024-65613-0 38926487 PMC11208546

[B118] Zhou K. Li Y. J. Soderblom E. J. Reed A. Jain V. Sun S. (2023). A “best-in-class” systemic biomarker predictor of clinically relevant knee osteoarthritis structural and pain progression. Sci. Adv. 9 (4), eabq5095. 10.1126/sciadv.abq5095 36696492 PMC9876540

