# Supplementary material for: Artificial intelligence in the biomechanics of knee osteoarthritis: biomechanical phenotyping, risk stratification, diagnosis, treatment and clinical translation—a narrative review
Source: Front Bioeng Biotechnol. 2026 Sep 24;14:1937199. doi: 10.3389/fbioe.2026.1937199 (PMC13647466; doi:10.3389/fbioe.2026.1937199)
Supplement: Supplementary file 1 [file Supplementaryfile1.docx]

Supplementary Material

# 1 Supplementary Material 1: Database-specific search strategies and record counts.

The search dates range from January 1, 2010, to July 1, 2026. The databases searched are China National Knowledge Infrastructure (CNKI), PubMed, and the Web of Science Core Collection. The search strategy combines terms related to knee osteoarthritis with those related to artificial intelligence, machine learning, biomechanics, gait, wearable sensing, element finite analysis, digital twin, diagnosis, prediction, treatment, and rehabilitation.

**CNKI**

Database: China National Knowledge Infrastructure (CNKI).

Search field: Subject/Topic.

Search result: n = 461.

Search string:

主题 =（膝骨关节炎 OR 膝关节骨关节炎 OR 膝骨性关节炎）AND 主题 =（人工智能 OR 机器学习 OR 深度学习 OR 影像组学 OR 神经网络 OR 可穿戴 OR 步态 OR 生物力学 OR 有限元 OR 数字孪生 OR 诊断 OR 治疗 OR 康复 OR 预测）

**PubMed**

Database: PubMed.

Search result: n = 600.

Search string:

("knee osteoarthritis"[Title/Abstract] OR "knee osteoarthrosis"[Title/Abstract] OR "Osteoarthritis, Knee"[MeSH Terms]) AND ("artificial intelligence"[Title/Abstract] OR "machine learning"[Title/Abstract] OR "deep learning"[Title/Abstract] OR radiomics[Title/Abstract] OR "neural network"[Title/Abstract] OR "wearable sensor"[Title/Abstract] OR gait[Title/Abstract] OR biomechanics[Title/Abstract] OR "finite element"[Title/Abstract] OR "digital twin"[Title/Abstract] OR diagnosis[Title/Abstract] OR prediction[Title/Abstract] OR rehabilitation[Title/Abstract] OR treatment[Title/Abstract])

**Web of Science Core Collection**

Database: Web of Science Core Collection.

Search result: n = 1857.

Search string:

TS = ("knee osteoarthritis" OR "knee osteoarthrosis" OR "osteoarthritis of the knee") AND TS = ("artificial intelligence" OR "machine learning" OR "deep learning" OR radiomics OR "neural network*" OR "wearable sensor*" OR gait OR biomechanic* OR "finite element" OR "musculoskeletal model*" OR "digital twin*" OR diagnosis OR prediction OR rehabilitation OR treatmen

# 2 Supplementary Material 2.

Flow diagram of literature identification and selection.

# 3 Supplementary Material 3: English annotations of Chinese-language sources included in the review.

| **Reference** | **Original Chinese title** | **English title** | **Source language / database** | **Design / sample** | **Main methods** | **Main findings** | **Limitations and evidence boundary** |
| --- | --- | --- | --- | --- | --- | --- | --- |
| **Guo et al., 2025** | 有限元分析技术在膝关节生物力学应用中的特点与优势 | Characteristics and advantages in finite element analysis techniques in knee biomechanics | Chinese; English abstract available. CNKI | Structured narrative review. PubMed, EI, CNKI, and Wanfang were searched in April 2024; 75 publications were included. | Synthesized methods for constructing knee finite-element (FE) models and their applications to osteoarthritis mechanisms, rehabilitation/physical interventions, surgical planning, implant design, and AI-assisted image segmentation and model construction. | FE analysis can visualize internal stress/strain and load redistribution, support mechanistic interpretation of cartilage degeneration, assess load-modifying interventions, and assist preoperative simulation and implant optimization. The review also describes AI-assisted segmentation and automated or semi-automated modeling as potentially improving efficiency and reproducibility. | The reviewed FE evidence is based on digital simulation and often simplifies ligaments, muscles, fat, and other soft tissues; validation standards are inconsistent; many models are derived from young or otherwise narrow populations; and the workflow remains technically demanding. This source provides mechanistic and implementation context, not direct evidence of AI clinical efficacy. |
| **Liu et al., 2024** | 不同骨密度对膝关节单髁置换后关节内各结构影响的三维有限元分析 | Three-dimensional finite element analysis of effects of different bone densities on various intra-articular structures after unicompartmental knee arthroplasty | Chinese; English abstract available. CNKI | Original computational biomechanical study based on CT and MRI data from one volunteer. Three postoperative models represented normal bone density, osteopenia, and osteoporosis. | A normal knee model was created with Mimics, Geomagic Studio, and ANSYS Workbench. Material properties were modified to generate osteopenic and osteoporotic models. Fixed-bearing medial unicompartmental knee arthroplasty was simulated, and a 1,000-N vertical load was applied. Stress and strain in the meniscus, tibial cartilage, polyethylene insert, cortical bone, and cancellous bone beneath the tibial component were compared. | The osteoporotic model showed higher peak stress in the meniscus and polyethylene insert, lower tibial-cartilage stress, increased cortical-bone stress near the medial edge, reduced cancellous-bone stress, and greater insert and cancellous-bone strain. The authors inferred that osteoporosis may contribute to abnormal postoperative load transfer and potentially increase loosening, periprosthetic fracture, and revision risk. | Single-subject geometry; body mass and sex were not modeled; only static extension loading was simulated; possible micromotion at interfaces was ignored; cartilage and meniscus were assigned simplified material properties; and all components were placed in standard positions. Findings are simulation-based and require clinical validation. |
| **Xie et al., 2023** | 有限元建模和分析在膝骨关节炎退变中的研究进展 | Research progress on finite element modeling and analysis of knee osteoarthritis degeneration | Chinese; English abstract available. CNKI | Narrative review; no reproducible database-specific search strategy, screening process, or included-study count was reported. | Reviewed FE approaches modeling articular cartilage, subchondral bone, coupled cartilage–bone degeneration, meniscal/musculoskeletal mechanics, and anterior cruciate ligament reconstruction. The article compared modeling assumptions and discussed their use for predicting structural degeneration and mechanically vulnerable regions. | FE models can integrate tissue mechanics and loading to explore knee-osteoarthritis progression, reproduce selected cartilage and subchondral-bone degeneration mechanisms, evaluate meniscal injury, and identify regions potentially susceptible to degeneration after ligament reconstruction. The review advocates integrating complementary models into a more powerful open computational framework for personalized risk assessment. | The review did not report a systematic search or study-selection method. The underlying models represent only subsets of OA biology, frequently simplify tissue composition and damage mechanisms, and vary substantially in assumptions and validation. The source supports mechanistic hypothesis generation rather than clinical effectiveness or causal claims. |
| **Yang et al., 2025** | 机器人辅助全膝关节置换术治疗膝骨关节炎内翻畸形的早期疗效 | Early impact of robot-assisted total knee arthroplasty on the treatment of varus knee osteoarthritis | Chinese; English abstract available. CNKI | Single-center retrospective comparative cohort of 59 patients with severe varus knee osteoarthritis undergoing total knee arthroplasty: robot-assisted group, n=28; conventional group, n=31. Follow-up was 6–8 months. | Compared operative time, incision length, blood loss, time to ambulation, complications, range of motion, Knee Society Score, visual analogue pain score, hip–knee–ankle angle, distal femoral and proximal tibial angles, and postoperative component-alignment measures. | Robot-assisted surgery required longer operative time and a longer incision but was associated with less blood loss, earlier ambulation, and more favorable selected component-alignment measures. Knee Society and pain scores were better in the robot group at 1, 3, and 6 months; range of motion was better at 1 and 3 months but not at 6 months. No major complications were reported in either group. | Retrospective, non-randomized design; small sample; short follow-up; uncertain long-term benefit; and possible operator/planning and learning-curve effects. Robotic assistance should be treated as digital surgical implementation context unless a substantive AI analytical, predictive, or adaptive function is demonstrated. |
| **Zhang et al., 2026** | 膝内侧间室骨关节炎单髁置换中有限元分析的应用价值 | Application value of finite element analysis in unicompartmental knee arthroplasty for medial-compartment knee osteoarthritis | Chinese; English abstract available. CNKI | Structured review. PubMed and CNKI were searched from database inception to December 2024; 53 publications were included. | Synthesized FE studies of tibial and femoral component alignment, joint-line restoration, bearing and tibial-tray design, and periprosthetic fracture risk in unicompartmental knee arthroplasty for medial-compartment osteoarthritis. | The review proposed parameter ranges for fixed- and mobile-bearing tibial and femoral component alignment, recommended maintaining the native joint line, and summarized design features potentially associated with more favorable stress distribution. It positions FE analysis as a tool for preoperative planning, implant optimization, and biomechanical risk assessment. | Substantial heterogeneity in model construction, material assignment, boundary conditions, and loading limits direct comparison. Patient-level factors such as age, body mass, and bone density were incompletely represented. Most analyses were static and did not fully model ligament balance, osteoporosis, or dynamic stability; clinical validation and translation remain limited. This is non-AI computational context. |
| **Yang et al., 2023** | 功能学对线机器人辅助膝关节置换术研究进展 | Research progress of functional alignment in robot-assisted knee arthroplasty | Chinese; English abstract available. CNKI | Narrative review; no explicit database search, screening method, or included-study count was reported. | Compared mechanical, kinematic, and functional alignment concepts and summarized robot-assisted functional-alignment total knee arthroplasty, including restoration of native joint-line orientation, component positioning, soft-tissue balance, early clinical outcomes, and implementation constraints. | Functional alignment seeks to combine patient-specific alignment with balanced flexion–extension gaps and preservation of the soft-tissue envelope. Early observational evidence suggests improved soft-tissue balance and patient-reported outcomes, but evidence remains preliminary. The review also highlights longer operative time, limited availability, and higher implementation cost. | Most cited functional-alignment studies were short-term and non-randomized; long-term implant survival and safety are uncertain. Longer operative time may increase procedural burden, and access is limited by robot availability and cost. Robotic surgery is not AI by default and should not be interpreted as direct evidence of AI efficacy. |
| **Xie, 2021** | 基于互联网的膝骨关节炎远程康复的随机对照临床试验 | A randomized controlled trial of internet-based rehabilitation for patients with knee osteoarthritis | Chinese; English abstract available. CNKI | Two-part master's thesis: (1) systematic review of 6 randomized trials, with 4 trials (791 participants) included in meta-analysis; and (2) a 12-week, single-blind, community-based randomized controlled trial of 56 patients allocated 1:1 to internet-based telerehabilitation or conventional rehabilitation; 49 completed follow-up. | The trial combined the self-developed 'For KOA' app and an Internet-of-Things activity wristband with education, self-management, cycling/strength exercise, and identical medication in both groups. Feasibility, adverse events, attrition, satisfaction, 11-point pain score, WOMAC pain/function, and SF-36 were assessed at baseline, 6 weeks, and 12 weeks using intention-to-treat analysis. | The meta-analysis found a small pain benefit versus conventional rehabilitation (SMD −0.21, 95% CI −0.40 to −0.01) but no significant functional benefit. In the trial, no adverse events were reported; attrition was 12.5%. Internet-based rehabilitation produced greater improvement in the 11-point pain score and selected SF-36 domains, whereas WOMAC physical-function improvement was similar between groups. | Meta-analysis: few eligible studies, heterogeneous outcomes, no postsurgical populations, no moderator analysis, and limited long-term follow-up. Trial: modest sample, 12-week follow-up, no validation of wristband measurement accuracy, no stratification by sex, education, income, or radiographic severity, and a relatively long app-learning period that may have contributed to attrition. The intervention was digital telerehabilitation, not necessarily AI. |

*Abbreviations: AI, artificial intelligence; CNKI, China National Knowledge Infrastructure; FE, finite element; KOA, knee osteoarthritis; TKA, total knee arthroplasty; WOMAC, Western Ontario and McMaster Universities Osteoarthritis Index.*

# 4 Supplementary Material 4: Structured characteristics of studies summarized in Tables 1–5.

| **Study** | **Sample size** | **Cohort source** | **Study design** | **AI method** | **Input modality** | **Outcome definition** | **Validation approach** | **Main performance metrics** |
| --- | --- | --- | --- | --- | --- | --- | --- | --- |
| **Table 1. Imaging AI for structural phenotyping, tissue segmentation, and severity recognition** | | | | | | | | |
| **Dang et al., 2026**  *MRI-based radiomics to determine the role of thigh muscle quality in the development of knee osteoarthritis: A discovery and validation study* | 448 OAI participants without radiographic KOA at baseline: 224 incident cases and 224 matched controls. Training n=314 (157/157); internal test n=134 (67/67). | Pivotal OAI Magnetic Resonance Imaging Analyses (POMA) incident-OA cohort, derived from the prospective multicenter Osteoarthritis Initiative (four US clinical centers); baseline to 48 months. | Nested case-control prognostic modeling study within a prospective multicenter cohort; cases and controls matched 1:1 by age, sex, and contralateral-knee status. | Radiomics pipeline for extensor, flexor, and auxiliary muscle groups: reproducibility filtering (ICC>0.80), mRMR ranking, LASSO logistic regression, muscle-specific Rad-scores, and multivariable logistic-regression fusion model. | Baseline axial T1-weighted thigh MRI for discovery; axial MPR 3D DESS water-excitation knee MRI for cross-sequence/anatomic-plane validation; conventional muscle CSA, intramuscular adipose-tissue CSA, and clinical variables. | Incident radiographic KOA, defined as Kellgren-Lawrence grade >=2 at any annual assessment within 48 months among participants with baseline KL grade <=1. | Participant-level random 7:3 training/test split. The same participants and muscle groups were reanalyzed on a different MRI sequence and anatomic plane as a validation procedure. Calibration, decision-curve analysis, subgroup analyses, and DeLong comparisons were reported. No independent external cohort. | Extensor Rad-score test AUC: 0.832 (95% CI 0.765-0.899) in the thigh-MRI discovery analysis and 0.807 (0.734-0.879) in the knee-MRI validation analysis. It exceeded extensor CSA (0.595/0.587), intramuscular-fat CSA (0.600/0.637), and the clinical model (0.577 in both test analyses). The fusion model did not provide a clearly greater net benefit than the extensor Rad-score alone. |
| **Yao et al., 2026**  *Deep learning-enabled segmentation of knee cartilage in conventional magnetic resonance images: Internal and external validation of different models* | 254 knees from 254 patients. Center 1: 219 knees, divided into training n=163 and internal validation n=56. Center 2: independent external validation n=35. | Retrospective MRI cohorts from two radiology centers in China. Center 1 data: 2017-2019 and April 2021; Center 2 data: April-November 2022. | Retrospective two-center development and external-validation segmentation study. | Three volumetric convolutional segmentation models: 3D Res U-net (primary), 3D U-net, and 3D V-net. Manual labels were created independently by two musculoskeletal radiologists. | Conventional sagittal proton-density fat-saturated knee MRI from 3.0-T scanners; N4 bias correction, cropping, intensity normalization, and isotropic resampling. | Automated segmentation of lateral femorotibial, medial femorotibial, and patellofemoral cartilage against expert manual masks; regional volume agreement also assessed. | Random internal training/validation split at Center 1 plus an independent external cohort from Center 2. Performance assessed using Dice, Jaccard/IoU, average and 95th-percentile Hausdorff distance, ICC, and Bland-Altman analysis. | 3D Res U-net Dice in internal validation: 0.871 (lateral tibiofemoral), 0.860 (medial tibiofemoral), and 0.858 (patellofemoral); external Dice: 0.846, 0.837, and 0.819. External Jaccard: 0.736, 0.723, and 0.699; external HD95: 1.052, 1.223, and 1.220 mm, respectively. |
| **Mu et al., 2026**  *Total knee radiomic feature atlas derived from magnetic resonance imaging segmentation: Insights into osteoarthritis incidence predictors* | KOA-onset prediction cohort: 386 propensity-matched OAI participants (193 incident KOA; 193 non-occurrence), split 7:3 into development n=270 and test n=116. Segmentation cohort was reported as 506 OAI patients; however, the listed components (193+193+122) total 508. | Osteoarthritis Initiative (OAI), a prospective multicenter cohort. Baseline KL<2; right-knee MRI used. Incident and non-incident participants matched 1:1 by age, race, sex, and BMI. | Nested case-control analysis of a prospective cohort, combining automated whole-knee segmentation, radiomic biomarker discovery, prognostic modeling, and a multi-reader assistance experiment. | 3D nnU-Net segmentation of 29 knee structures; 3,219 radiomic features per participant; variance testing, correlation filtering, LASSO, fivefold cross-validated feature ranking, and logistic-regression Radkneeatlas models. | Baseline sagittal 3D DESS knee MRI; 29 segmented bone, cartilage, meniscal, ligament, and effusion structures; radiomic and morphologic features. | Four-year incident radiographic KOA, defined as progression from baseline KL<2 to KL>=2. Secondary evaluation measured whether Radkneeatlas assistance improved junior surgeons' predictions. | Segmentation dataset randomly split 8:2. Prediction dataset split 7:3 at participant level; feature selection restricted to development data with fivefold cross-validation. Calibration plots, DeLong testing, and a crossover multi-reader study were performed. No independent external cohort. | Mean Dice across 29 structures: 0.958. Optimized 18-feature Radkneeatlas model: AUC 0.918 (95% CI 0.841-0.954), specificity 0.882, precision/PPV 0.893, recall 0.769, F1 0.826, accuracy 0.819, and NPV 0.750. Junior-surgeon mean accuracy increased from 0.605 to 0.813 with AI assistance. |
| **Li et al., 2026**  *ERANet: Edge replacement augmentation for semi-supervised meniscus segmentation with prototype consistency alignment and conditional self-training* | OAI-iMorphics DESS dataset: 176 examinations (training n=140; test n=36). Independent in-house FSE dataset: 33 OA patients (training n=27; test n=6). Semi-supervised experiments simulated limited annotation, including as little as 10% labeled data. | Public OAI-iMorphics MRI dataset and an institutional dataset acquired at Prince of Wales Hospital, Hong Kong, in 2021 (KL grades 1-4). | Method-development and comparative semi-supervised segmentation study evaluated separately on two datasets with different MRI pulse sequences and contrast characteristics. | ERANet mean-teacher framework integrating edge replacement augmentation, prototype consistency alignment, conditional self-training, temporal-stability filtering of pseudo-labels, and four sequential segmentation networks. | Sagittal 3D DESS MRI (OAI-iMorphics) and 3D FSE/TSE MRI (in-house); manually annotated lateral and medial menisci, with labeled training scans partially treated as unlabeled. | Pixel-wise segmentation of lateral and medial menisci against expert manual masks under limited-label conditions. | Random train/test division within each dataset; experiments repeated across several labeled/unlabeled ratios; comparison with multiple state-of-the-art semi-supervised methods and extensive ablation analyses. The second dataset tested reproducibility across another sequence, but the model was trained and evaluated separately rather than externally transferred across datasets. | OAI-iMorphics: lateral/medial/mean Dice 88.86%/84.46%/86.66%; mean ASSD 0.33 mm. In-house FSE: Dice 78.17%/56.01%/67.09%; mean ASSD 3.36 mm. ERANet achieved the highest mean Dice among compared semi-supervised methods on both datasets. |
| **Lau et al., 2025**  *Diffusion model-empowered patella shape analysis predicts knee osteoarthritis outcomes* | 2,913 MOST participants with baseline radiographs; patient-level training n=2,330 and test n=583. Diffusion-model subset with paired baseline/60-month radiographs: n=1,953 (1,565/388). Patella-segmentation subset: 1,460 images (1,168/292). | Multicenter Osteoarthritis Study (MOST); left weight-bearing lateral knee radiographs at baseline and 60 months, with KL grade, patellofemoral OA, knee-replacement events, and baseline WORMS data. | Retrospective longitudinal cohort analysis with automated segmentation, generative trajectory modeling, and prognostic classification. | Two-step attention U-Net/ResNeSt-101 patella segmentation; conditional denoising diffusion probabilistic model to generate synthetic 60-month patella shapes; SynPatNet two-channel 1D CNN using baseline plus synthetic follow-up shapes. | Baseline lateral knee radiographs and automated baseline patella masks; paired 60-month masks for diffusion training; synthetic follow-up shape; KL grade and baseline patellofemoral OA for comparator/combined models. | Within 60 months: (1) incident patellofemoral OA by MOST radiographic criteria; (2) incident tibiofemoral OA from KL<2 to KL>=2; and (3) any knee arthroplasty. | Consistent 8:2 patient-level training/test split throughout the pipeline to avoid leakage. Test-set AUC and PR-AUC with 95% CIs; DeLong tests; diffusion image-quality and overlap metrics. No independent external cohort. | Diffusion test performance: Dice 0.927, IoU 0.867, SSIM 0.915, MSE 0.028, PSNR 16.21 dB, LPIPS 0.079. SynPatNet AUC: 0.909 for patellofemoral-OA onset, 0.597 for tibiofemoral-OA onset, and 0.823 for knee replacement. KL grade plus SynPatNet achieved AUC 0.838 versus 0.785 for KL alone. |
| **Chavoshi et al., 2026**  *Comparative Evaluation of Deep Learning and Foundation Model Embeddings for Osteoarthritis Feature Classification in Knee Radiographs* | 44,985 unilateral knee radiographs from 4,508 OAI participants. | Osteoarthritis Initiative radiographic dataset with expert OARSI feature grades and KL grades; images cropped using the DUKE knee-localization model. | Retrospective comparative classification study of task-specific supervised deep learning versus pretrained foundation-model embeddings. | ImageNet-pretrained ResNet18 and ConvNeXt-Small fine-tuned end to end; BiomedCLIP and RAD-DINO frozen embeddings classified with XGBoost; additional BiomedCLIP image-plus-structured-text experiment; Grad-CAM for qualitative interpretability. | Knee radiographs after percentile intensity scaling, automated cropping, CLAHE, and augmentation; structured templated radiology text added for the multimodal BiomedCLIP analysis. | Multiclass KL grade 0-4; binary presence of medial/lateral osteophytes, joint-space narrowing, subchondral sclerosis, and compartmental OA, with feature presence defined as OARSI grade >=2. | Stratified patient-level split: 80% training, 10% validation, 10% test. Test metrics reported with 1,000-iteration bootstrap 95% CIs. No independent external cohort; Grad-CAM review was qualitative only. | KLG classification: ConvNeXt weighted kappa 0.880 (95% CI 0.871-0.889), accuracy 0.704; ResNet18 kappa 0.874, accuracy 0.694. BiomedCLIP image+text: kappa 0.551, accuracy 0.512; RAD-DINO: 0.521/0.488. ConvNeXt binary AUC examples: medial/lateral subchondral sclerosis 0.892/0.865. BiomedCLIP zero-shot knee-radiograph identification: 91.14%. |
| **Park et al., 2026**  *Enhanced Magnetic Resonance Imaging-Based Knee Cartilage Segmentation Using a Swin-UNet Conditional Generative Adversarial Network: Development and Validation Study* | Internal dataset: 232 KOA MRI scans (training n=162; validation n=35; test n=35). Independent external validation: 25 consecutive PSI-guided TKA cases. | Yonsei Sarang Hospital internal cohort (October 2023-December 2024) and Yongin Severance Hospital external cohort; patients undergoing patient-specific-instrumentation-guided TKA. | Retrospective two-center model-development and external-validation segmentation study. | Swin-UNet conditional GAN with hierarchical Swin-UNet generator and pixel-wise CNN discriminator; compared with UNet, UNet cGAN, and Swin-UNet. | Internal: sagittal 3D high-resolution T2*-weighted gradient-echo knee MRI at 3.0 T. External: independent 3.0-T knee MRI acquired on a different scanner/protocol. Manual femoral and tibial cartilage masks. | Automated femoral and tibial cartilage segmentation against manual ground truth for potential MRI-based surgical planning. | Case-level 70%/15%/15% internal split plus independent external hospital dataset. DSC, IoU, ASSD, and HD95; intra- and interrater label reliability; ablation study and inference-time analysis. | Internal test: femoral DSC 82.0%, IoU 71.9%, ASSD 0.22 mm, HD95 1.31 mm; tibial DSC 81.0%, IoU 71.1%, ASSD 0.23 mm, HD95 1.25 mm. External: femoral DSC 73.1%, IoU 65.0%, ASSD 1.30 mm, HD95 5.91 mm; tibial DSC 69.7%, IoU 62.6%, ASSD 2.16 mm, HD95 7.18 mm. Mean inference time: 1.68 s per patient volume. |
| **Table 2. Gait AI, wearable sensing, and real-world load assessment** | | | | | | | | |
| **Mapinduzi et al., 2026**  *Wearable Sensors and Artificial Intelligence for Ecological Knee Osteoarthritis Assessment: Development and Feasibility of a Hybrid Digital Phenotyping Framework* | 40 participants: 20 patients with bilateral knee OA and 20 age-matched healthy controls. | Patients were recruited from the outpatient rheumatology clinic of the Institut National de Santé Publique, Bujumbura, Burundi (September 2024-January 2025); controls were recruited through community advertisements from the same geographic area. | Perspective/framework article incorporating a proof-of-concept, cross-sectional feasibility pilot. The pilot compared multidimensional functional and sensor-derived variables between knee OA and control groups. | Machine-learning-ready data-integration framework rather than a trained diagnostic AI model. The implemented pipeline used predefined missing-data handling, bilateral-variable aggregation, z-score normalization, hierarchical clustering, heatmap visualization, and Pearson correlation analysis; anomaly detection, cloud storage, and future supervised, unsupervised, and reinforcement-learning analyses were proposed. | Questionnaires and clinical assessments (including pain, WOMAC, kinesiophobia, quality of life, physical activity, range of motion, strength, balance, 6-minute walk, and sit-to-stand), connected insoles, force plates, electronic goniometry and dynamometry, Fibion activity sensing, Polar smartwatch data, and seven-day home monitoring. | Feasibility of harmonizing supervised clinical/laboratory assessments with unsupervised real-world monitoring, and exploratory identification of variables differentiating bilateral knee OA from healthy controls. No prespecified AI diagnostic, prognostic, or treatment-response endpoint was evaluated. | Proof-of-concept evaluation in the 40-participant pilot using exploratory group comparisons, within-group hierarchical clustering, and variable-group correlations. No trained predictive model, held-out AI test set, or independent validation cohort was reported. | No AI classification or prediction metric was reported. Exploratory correlations with OA status were strongest for gait speed (r=-0.698), stride length (r=-0.680), loading phase (r=-0.570), cadence (r=-0.552), and stance-phase duration (r=0.537). Findings were explicitly described as hypothesis-generating. |
| **Snyder et al., 2025a**  *Prediction of knee loads during activities of daily living using custom instrumented insoles and machine learning* | 47 healthy young adults (23 female, 24 male; mean age 23.4±3.9 years). Analytic data included 462 walking strides, 429 running strides, 228 sit-to-stand trials, and 228 stand-to-sit trials. | Single-center laboratory cohort recruited for a biomechanics study at the University of Maryland, College Park, USA. Participants had no major lower-limb injury, lower-extremity surgery, or mobility problem affecting gait. | Laboratory-based model-development study. Participants completed walking, running, sit-to-stand, and stand-to-sit tasks while wearable-insole, motion-capture, force-plate, and ultrasound data were collected. | Activity- and outcome-specific recurrent neural networks. CNN, multilayer perceptron, and RNN candidates were compared; the selected model used an LSTM, ReLU, dropout, flatten, and linear output layer. Twelve separate models were developed for four activities and three knee-load outcomes. | Right-foot custom instrumented insole with five 6-degree-of-freedom force/moment sensors; the models used 15 force channels normalized to 101 time points per activity cycle. Motion capture and force plates supplied inverse-dynamics reference moments, and ultrasound-informed reduction modeling supplied medial joint contact force labels. | Continuous waveform estimation, plus exploratory peak estimation, of medial joint contact force (MJCF), knee flexion moment (KFM), and knee adduction moment (KAM) during walking, running, sit-to-stand, and stand-to-sit activities. | Participant-level random split: 70% training, 13% validation, and 17% held-out testing, with each participant confined to one subset. Predictions were compared with laboratory references using Pearson correlation and mean absolute error. No external cohort validation was performed. | MJCF waveform r=0.88-0.98 with MAE 0.27-0.41 BW; KFM r=0.89-0.98 with MAE 0.09-0.18 Nm/kg; KAM r=0.48-0.94 with MAE 0.37-0.46 %BW×Ht. KAM performance was strongest for walking (r=0.93) and running (r=0.94), but only moderate for sit-to-stand (r=0.50) and stand-to-sit (r=0.48). |
| **Feng et al., 2025**  *Self-Aware Fusion IMU-EMG Attention Dependence for Knee Adduction Moment Estimation During Walking* | 18 effective participants. | Recruitment setting, institution, participant clinical status, and demographic characteristics were not reported in the supplied abstract. | Laboratory-based deep-learning method-development study using walking data to estimate knee adduction moment. | Self-aware multimodal fusion deep-learning framework with attention-dependent integration of inertial measurement unit and electromyography signals. | Walking signals from four inertial measurement units and six electromyography channels. | Estimation of stance-phase knee adduction moment, described as a biomechanical measure relevant to knee OA severity. The supplied abstract did not report the reference-standard acquisition method or whether the target was a waveform, peak, or summary value. | Compared with counterpart estimation methods. Data partitioning, cross-validation procedure, held-out testing, and external validation were not reported in the supplied abstract. | The proposed model reportedly reduced relative root-mean-square error by 9.15% BW·BH compared with counterpart estimation methods. Additional metrics and uncertainty estimates were not reported in the supplied abstract. |
| **Moura et al., 2025**  *Knee osteoarthritis prediction from gait kinematics: Exploring the potential of deep neural networks and transfer learning methods for time series classification* | 54 participants: 27 patients with knee OA and 27 asymptomatic healthy individuals. | Recruitment setting, institution, diagnostic criteria, and participant demographics were not reported in the supplied abstract. | Cross-sectional case-control diagnostic classification and model-comparison study based on lower-limb gait-kinematic time series. | Joint-angle time series were transformed into color-based images, recurrence plots, and Gramian angular fields. A three-convolutional-layer baseline network identified the Gramian Angular Difference Field representation, after which 10 pretrained deep neural networks were compared using transfer learning and fine-tuning; VGG16 and Xception performed best. | Lower-limb joint-angle time series acquired during gait and encoded as image representations, with the final models using Gramian Angular Difference Field images. | Binary classification of participants as knee OA patients or asymptomatic healthy controls. | Comparative evaluation of 10 transfer-learning architectures on the GADF dataset. Training/test partitioning, cross-validation, confidence intervals, and external validation were not reported in the supplied abstract. | Classification accuracy was 89.7% for VGG16 and 92.3% for Xception. Sensitivity, specificity, AUC, calibration, and uncertainty estimates were not reported in the supplied abstract. |
| **Table 3. Computational modeling, finite-element integration, and digital-twin applications** | | | | | | | | |
| **Paz et al., 2024**  *Knee-Loading Predictions with Neural Networks Improve Finite Element Modeling Classifications of Knee Osteoarthritis: Data from the Osteoarthritis Initiative* | 97 knees from 97 OAI participants (71% female) with a radiographically healthy baseline and 8-year follow-up data. | Osteoarthritis Initiative (OAI), a United States multicenter longitudinal cohort. Baseline radiographs, MRI, demographic and walking data were linked to radiographic status after 8 years. | Retrospective longitudinal computational-modeling and classification study. Template-based medial and lateral tibiofemoral finite-element models were generated for each knee and evaluated under alternative loading, cartilage-thickness, and tissue-failure assumptions. | Previously developed feed-forward artificial neural networks estimated medial, lateral, and total peak knee joint contact forces during stance from simple subject characteristics. Neural-network load estimates were coupled to individualized template-based finite-element simulations and age-dependent maximum-principal-stress degeneration criteria. | Height, weight, age, sex, self-selected walking speed, frontal-plane knee alignment, and simple MRI-derived knee dimensions/cartilage-thickness measurements; baseline radiographs and 8-year radiographs supplied Kellgren-Lawrence grade and joint-space-width data. A generic gait-load waveform was scaled using neural-network estimates. | Primary modeling output: percentage cartilage volume exceeding an age-dependent tensile-stress threshold during stance. Clinical classification target: future radiographic OA group at 8 years (KL 0-1, KL 2, or KL 3-4). Joint-space narrowing from baseline to 8 years was used as a secondary verification outcome. | The study compared three joint-contact-force definitions, two cartilage-thickness scaling approaches, and multiple degeneration thresholds. Classification was assessed by ROC AUC with DeLong testing in the same 97-knee cohort; simulated degeneration was compared with compartmental joint-space narrowing. No independent held-out or external cohort was used in this study. The previously developed neural networks had correlations of r=0.61±0.15, 0.67±0.13, and 0.80±0.10 for medial, lateral, and total peak contact force, respectively. | The best configuration—neural-network-predicted compartmental loading with the template femur-to-tibia thickness ratio—had a median AUC of 0.70 (range 0.50-0.78 across compartments and failure thresholds). No configuration discriminated KL 0-1 from KL 2. Neural-network loading produced a median 2.6-fold higher simulated degeneration in the medial than lateral compartment (p<0.001). Association with measured joint-space narrowing was weak (adjusted R²=0.104). |
| **Hoyer et al., 2025**  *Foundations of a Knee Joint Digital Twin from qMRI Biomarkers for Osteoarthritis and Knee Replacement* | OAI source cohort: 4,796 participants and 59,836 knee MRI examinations across 10 years; 4,283 participants remained after baseline data cleaning. Matched analyses included 357 incident-OA cases and 319 controls, and 253 knee-replacement cases and 233 controls. | Osteoarthritis Initiative (OAI), a United States multicenter longitudinal imaging cohort with baseline and follow-up radiographs, bilateral 3D-DESS and 2D-MSME MRI, demographic variables, KOOS/WOMAC measures, and knee-replacement records. | Retrospective matched-cohort imaging-biomarker study establishing the analytical foundation for a knee digital-twin framework. The reported analyses were primarily cross-sectional comparisons of baseline qMRI features according to subsequent OA incidence or knee replacement, rather than a continuously updating clinical digital twin. | Ensembles of 2D and 3D V-Net models automatically segmented bone, cartilage, and menisci. Cartilage thickness, cartilage T2 relaxation, bone shape, and meniscus shape were reduced to a 110-dimensional principal-component feature space. t-SNE plus nearest-neighbor matching balanced outcome and control cohorts; bootstrap elastic-net stability selection and binomial generalized linear models identified outcome-associated biomarkers. | Baseline sagittal 3D-DESS MRI, 2D multi-slice multi-echo MRI for cartilage T2 mapping, radiographs, derived 3D bone/cartilage/meniscus surfaces, and demographic and clinical variables including age, sex, BMI, KOOS, WOMAC, pain, and functional measures. | OA-incidence analysis: development of radiographic OA after a baseline KL grade <2 versus a clinically matched non-incident control. Knee-replacement analysis: subsequent partial or total knee replacement versus a matched participant without replacement. Outputs were imaging-biomarker associations and odds ratios, not a deployed individual prediction score. | Segmentation was validated on an OAI subset; missing-data imputation was checked by sensitivity analysis. Cohort balance was assessed with standardized mean differences, Cohen d, Cramer V, point-biserial correlations, and paired nonparametric tests. Biomarker comparisons used Wilcoxon tests with Benjamini-Hochberg correction. Feature robustness was evaluated by elastic-net stability selection over 1,000 bootstrap samples followed by multivariable GLM. No external cohort, prospective temporal validation, calibration analysis, or clinical-utility evaluation was reported. | Segmentation Dice coeffcients were >0.85 for soft tissues and >0.95 for bone. Cross-sectional analysis identified 7 significant PC features for OA incidence and 38 for knee replacement. Representative multivariable associations included femoral cartilage-thickness PC1 (OR 0.973, 95% CI 0.959-0.987) and PC2 (OR 0.960, 95% CI 0.935-0.985) with lower OA-incidence odds; tibial bone-shape PC9 was associated with knee replacement (OR 1.038, 95% CI 1.016-1.059), whereas femoral cartilage-thickness PC2 was protective (OR 0.948, 95% CI 0.927-0.970). AUC and calibration metrics were not reported. |
| **Jayakumar et al., 2025**  *Shared Decision Making Using Digital Twins in Knee Osteoarthritis Care: A Randomized Clinical Trial of an AI-Enabled Decision Aid versus Education Alone on Decision Quality, Physical Function, and User Experience* | 222 patients were randomized (112 intervention; 110 control); 201 were included in the analytic sample (101 intervention; 100 control). The embedded AI/ML prediction module had been trained on a nationally representative dataset of >700,000 patients undergoing TKA. | Single university-affiliated orthopaedic clinic in the United States, February 2021-November 2022. Participants had advanced knee OA (KL grade 3-4) and were medically eligible for primary TKA. Trial registration: NCT04805554. | Randomized, open-label, stratified block clinical trial. The intervention included education, preference assessment, and a person-specific AI-derived TKA benefit-risk report; the control received the same education module alone. Outcomes were assessed after consultation and at 3 and 6-9 months. | Proprietary supervised AI/ML decision-support model combining regression and gradient-boosting frameworks. It generated person-specific probabilities of clinically meaningful benefit after TKA and complication risk, presented as a patient-level “digital twin” report alongside educational and preference information. | Age, sex, BMI, smoking status, Charlson Comorbidity Index, radiographic KL grade, baseline KOOS JR, PROMIS Global Mental Health score, and emergency-department visits and inpatient hospitalizations in the prior year; registry and health-system clinical and outcome data were used for model development. | Prediction-module targets: probability of achieving at least a minimal clinically important improvement in KOOS JR domains after TKA and probability of selected postoperative complications. Trial primary outcome: Knee OA Decision Quality Instrument process score. Secondary outcomes included CollaboRATE, decisional conflict, decision regret, KOOS JR, treatment concordance, satisfaction, consultation duration, and TKA use. | The underlying benefit and complication models were evaluated on independent validation datasets. Trial allocation used eight stratification cells and random blocks of 4 or 6; analyses followed the original assigned groups with prespecified endpoints, baseline adjustment where applicable, bootstrap confidence intervals, mixed-effects models for longitudinal outcomes, and Hochberg multiple-comparison adjustment. The clinical trial was single-center and unblinded after allocation; the proprietary model’s original development and validation were not independently reported in a separate peer-reviewed publication. | Underlying-model AUROC: 0.77 for TKA benefit and 0.72 for complications on independent validation data. In the trial, K-DQI was 84.4±25.2 versus 71.4±29.8 (adjusted difference 13.0, 95% CI 5.2-20.3; p=0.0011); decision conflict was 1.0±3.1 versus 3.3±5.8 (p=0.0029); 6-9-month decision regret was 18.2±19.5 versus 27.2±24.2 (p=0.0051); KOOS JR was 69.5±17.3 versus 47.0±18.4 (p<0.0001); and treatment concordance was 91.1% versus 76.0% (p=0.0043). TKA rate, consultation duration, satisfaction, and 3-month KOOS JR did not differ significantly. |
| **Lan et al., 2024**  *Reliable Prediction of Implant Size and Axial Alignment in AI-Based 3D Preoperative Planning for Total Knee Arthroplasty* | 60 patients with KOA undergoing primary TKA: 30 received AI-based 3D planning and 30 received conventional 2D radiographic template planning. | Department of Orthopaedics, Fujian Provincial Hospital, China; procedures performed from September 2021 to June 2023 by the same surgical team. All implants were from DePuy Synthes. | Single-center retrospective comparative cohort study of AI-based CT 3D preoperative planning versus conventional 2D X-ray templating, with postoperative radiographic assessment and clinical follow-up to 12 months. | AI-Knee software using a proprietary G-NET neural network to identify femur and tibia, detect landmarks, measure three-dimensional alignment, simulate tibial and femoral osteotomy, predict implant size and position, and generate simulated postoperative radiographs. | Preoperative knee CT in DICOM format plus full-length weight-bearing lower-limb radiographs. The comparator used anteroposterior/lateral knee radiographs, full-length radiographs, and manufacturer-supplied 2D prosthesis templates. | Planning outcomes: exact agreement between planned and implanted femoral/tibial component size, and postoperative femoral valgus correction angle (VCA) and hip-knee-ankle angle (HKA) outlier status. Clinical outcomes: WOMAC and American Knee Society scores before surgery and at 3, 6, and 12 months. | AI plans were verified against the implanted component sizes, full-length weight-bearing radiographs obtained 3 days postoperatively, and serial clinical scores. Results were compared with the contemporaneous 2D-planning group using t tests and chi-squared tests. No separate training, held-out test, cross-validation, or external validation cohort was reported. | Exact femoral and tibial size agreement was 90.0% (27/30) and 86.7% (26/30) with AI versus 66.7% (20/30) and 60.0% (18/30) with 2D planning (all p<0.05). VCA and HKA outlier rates were 13.3% and 3.3% with AI versus 53.3% and 30.0% with 2D planning. WOMAC and AKS outcomes were significantly better in the AI group at 3, 6, and 12 months (p<0.05), although both groups improved by 12 months. |
| **Table 4. Prediction, risk stratification, and treatment-response phenotyping** | | | | | | | | |
| **Zhou et al., 2023**  *A “Best-in-Class” Systemic Biomarker Predictor of Clinically Relevant Knee Osteoarthritis Structural and Pain Progression* | Discovery/derivation cohort: 596 participants with knee OA in the FNIH biomarker cohort (192 joint-space-loss [JSL] plus pain progressors, 103 JSL-only progressors, 102 pain-only progressors, and 199 nonprogressors). Independent validation cohort: 86 participants in the Biomarker Factory (BMF) cohort (37 radiographic progressors and 49 nonprogressors). | FNIH Osteoarthritis Biomarkers Consortium cohort, nested within the Osteoarthritis Initiative (OAI), with baseline serum and longitudinal radiographic and pain data. Independent BMF cohort with baseline serum and 3- to 4-year radiographic follow-up. | Prognostic biomarker discovery and independent validation study using a nested case-control derivation cohort. Baseline serum peptide biomarkers were evaluated for prediction of clinically relevant structural and pain progression over 48 months; the structural biomarker panel was subsequently tested in an independent cohort. | Targeted multiple-reaction-monitoring proteomics by UPLC-MS/MS. Bootstrapped elastic-net regression selected stable correlated peptide sets; backward elimination produced smaller essential biomarker panels. Multivariable logistic regression combined biomarker panels with clinical covariates and comparator urinary biomarkers. | Baseline serum peptide abundances: 107 quality-controlled peptides representing 64 proteins from an initial 177-peptide panel. Clinical variables included baseline Kellgren-Lawrence grade, WOMAC pain, sex, and other selected covariates. Comparator biomarkers included urinary CTX-II and urinary CTX-Iα. | Primary FNIH outcome: clinically relevant progression, defined as both radiographic JSL (minimum joint-space-width decrease ≥0.7 mm from 24 to 48 months) and sustained WOMAC pain worsening (increase ≥9/100 from 24 to 48 months, sustained at ≥2 follow-up visits). Secondary outcomes were JSL-only and pain-only progression. In BMF, radiographic progression was an increase of ≥1 categorical joint-space-narrowing grade over 3-4 years. | Feature stability and optimism were addressed with bootstrap resampling, elastic-net selection, backward elimination, multiple imputation, and bootstrapped ROC analysis. Performance was compared with clinical covariates and established urinary biomarkers. Generalizability was assessed in the independent BMF cohort; a family-relatedness sensitivity analysis randomly removed one member from each related pair. | Essential serum panels yielded AUCs of 0.728 for JSL-plus-pain progressors versus the composite comparator and 0.740 versus complete nonprogressors; JSL-only and pain-only AUCs were 0.698 and 0.673. The 13-peptide clinically relevant progression panel had 80.2% sensitivity and 59.3% specificity. The highest combined-model AUC was 0.794. Independent BMF validation of the 11-peptide structural panel yielded AUC 0.697 (0.688 after family sensitivity analysis), compared with approximately 0.58-0.61 for traditional comparator models. |
| **Alkhatatbeh et al., 2026**  *Clinical Features Outperform MRI Radiomics for Predicting Intra-Articular Hyaluronic Acid Response in Knee Osteoarthritis* | 262 participants/329 treated knees; MRI subset 106 participants/128 knees | OAI participants receiving intra-articular hyaluronic acid. | Retrospective treatment-response prediction and validation-design sensitivity study. | Clinical-only, DESS-MRI radiomics-only, and combined machine-learning models. | Baseline clinical variables and 3D DESS MRI radiomic features. | Study-defined clinical response to intra-articular hyaluronic-acid injection. | Repeated grouped participant-level 5x5 cross-validation; knee-level splitting evaluated as a sensitivity analysis for leakage. | Participant-level clinical model AUC 0.716 (95% CI 0.689-0.742); MRI-subset clinical AUC 0.718; radiomics AUC 0.499; knee-level combined AUC increased to 0.773, illustrating inflation from non-independent knees. |
| **Rockel et al., 2025**  *Deep Learning-Based Clustering for Endotyping and Post-Arthroplasty Response Classification Using Knee Osteoarthritis Multiomic Data* | 414 patients with symptomatic, radiographic KL grade III/IV primary KOA undergoing total knee arthroplasty. Deep-learning clustering produced cluster 1 (n=146), cluster 2 (n=138), and cluster 3 (n=130). | Longitudinal Evaluation in the Arthritis Program-OA cohort, University Health Network, Toronto, Canada. Plasma and urine were collected within 3 months before surgery, synovial fluid intraoperatively, and WOMAC outcomes at 1 year after TKA. | Single-cohort observational multiomic endotyping and treatment-response classification study. Unsupervised multimodal deep learning identified molecular endotypes, followed by cluster-specific supervised classification of 1-year pain and function response after TKA. | Novel omicVAE architecture with one fully connected encoder and four modality-specific decoders generated a shared latent space; K-means identified clusters. Differential expression and standardized mean-difference analyses identified signatures. Random-forest unimodal classifiers and a second-level multimodal meta-classifier integrated molecular and clinical domains for response classification. | Four preoperative molecular domains comprising 2,727 features: 151 plasma metabolites, 421 plasma microRNAs, 930 synovial-fluid microRNAs, and 1,225 urine microRNAs. Clinical inputs for response classification included age, sex, BMI, HADS anxiety/depression categories, and painDETECT category. Outcomes used baseline and 1-year WOMAC pain and function. | Endotyping outcome: three multimodal molecular clusters. Treatment-response outcomes: WOMAC pain and WOMAC function responders versus nonresponders at 1 year after TKA; response was defined as >33% improvement from baseline, whereas ≤33% improvement was nonresponse. | The number of clusters was supported by scree and silhouette analyses. Classification AUCs and 95% CIs were estimated using repeated internal cross-validation (reported as 10-time, 10-fold cross-validation). Integrated classifiers were compared with individual-domain models within each cluster. No independent external cohort was used. | Three endotypes were identified, with molecular signatures of 30, 16, and 24 features for clusters 1-3. Integrated pain-response AUCs were 0.841, 0.816, and 0.879 for clusters 1-3, exceeding the best unimodal AUCs of 0.735, 0.740, and 0.786. Integrated function-response AUCs were 0.786, 0.808, and 0.738; the best unimodal clinical-domain AUCs were 0.702, 0.791, and 0.722. |
| **Lee et al., 2025**  *Development of the Machine Learning Model That Is Highly Validated and Easily Applicable to Predict Radiographic Knee Osteoarthritis Progression* | Model development: 5,966 knees from the OAI. External validation: 3,392 knees from MOST. Baseline knees had Kellgren-Lawrence grades 0, 1, or 2; progression occurred in 668 OAI knees (11.2%) and 892 MOST knees (26.3%). | Osteoarthritis Initiative (OAI) for model development with 4-year follow-up; Multicenter Osteoarthritis Study (MOST) for independent external validation with 5-year follow-up. Both are large prospective multicenter observational cohorts. | Retrospective analysis of prospectively collected longitudinal cohort data, reported according to TRIPOD. A clinically deployable model was developed in OAI and evaluated without retraining in the distinct MOST cohort. | Logistic regression, k-nearest neighbors, gradient boosting, decision tree, random forest, and support-vector machine were compared; random forest achieved the best development AUC. Median imputation, hyperparameter tuning, and sequential feature selection were applied. SHAP values and partial-dependence plots supported interpretation, and a web-based calculator was created. | Fifteen candidate variables spanning age, sex, race, BMI, diabetes, Charlson comorbidity score, prior meniscectomy, hip-knee-ankle angle, medial and lateral joint-space-narrowing grades, baseline KLG, contralateral knee OA, WOMAC total and pain scores, and 20-m walking speed. The final model retained baseline KLG, contralateral OA, lateral JSN, BMI, medial JSN, and WOMAC total score. | Radiographic knee OA progression over 4 years in OAI and 5 years in MOST. Any increase in KLG was considered progression except KLG 0 to 1, which was prespecified as trivial and not counted as progression. | Internal model selection and tuning were performed in OAI. The finalized six-variable random-forest model was then tested in the fully independent MOST cohort. Validation included a confusion matrix, ROC analysis, accuracy, precision, recall, F1 score, and AUC. Feature effects were compared across cohorts by odds ratios, SHAP, and partial-dependence plots. | Development AUC was 0.75. Independent MOST validation yielded AUC 0.76, accuracy 0.68, precision 0.44, recall 0.77, and F1 score 0.56. Important predictors included lateral JSN (odds ratio 4.74), contralateral knee OA (OR 2.59), and baseline KLG (OR 1.76), all p<0.001. |
| **Table 5. Clinical translation, rehabilitation, and implementation** | | | | | | | | |
| **Wang et al., 2021**  *Sensor-Based Gait Retraining Lowers Knee Adduction Moment and Improves Symptoms in Patients with Knee Osteoarthritis: A Randomized Controlled Trial* | 89 patients were screened; 71 were randomized (walking exercise, n=36; sensor-based gait retraining, n=35). Sixty-two participants completed training and reassessment and were included in the per-protocol analysis (n=31 per group). | Patients with symptomatic early medial knee OA (Kellgren-Lawrence grade I or II), aged 40-70 years, were recruited from orthopaedic and trauma outpatient clinics in Hong Kong from January to August 2019. | Prospective, participant-blinded, parallel-group randomized controlled trial (HKUCTR-2487). Both groups completed six weekly treadmill sessions over 6 weeks; the intervention group received individualized real-time knee-loading feedback, whereas the comparator received time-matched walking exercise without biomechanical feedback. | A previously developed artificial neural network estimated the knee adduction moment (KAM) in real time from wearable inertial-sensor data and displayed the KAM curve on a tablet. Participants modified foot progression angle to target a 20% reduction in the KAM peak. The current article did not report the ANN architecture or retrain the model; it cited prior agreement with laboratory ground truth (R2=0.956). | Intervention input: a shoe-mounted lateral-ankle IMU containing a gyroscope (±2000 degrees/s) and accelerometer (±4 g), sampled at 100 Hz. Biomechanical outcome assessment used 10-camera motion capture (100 Hz) and a force plate (1000 Hz). Clinical inputs/outcomes included visual analogue pain score and KOOS/KOOS5 subscales. | Primary biomechanical and clinical outcomes were first and second KAM peaks (KAM1 and KAM2), immediate walking pain (VASP), and composite KOOS5 before and after the 6-week intervention. Secondary outcomes included knee adduction angular impulse (KAAI), knee flexion moment (KFM), foot progression angle, and individual KOOS pain, symptoms, activities-of-daily-living, sport/recreation, and quality-of-life subscales. | Computer-generated 1:1 allocation was concealed in sealed opaque envelopes; participants were blinded to group purpose. Clinical efficacy was evaluated per protocol using group-by-time mixed ANOVA with Bonferroni-adjusted comparisons and effect sizes. The RCT evaluated the AI-enabled feedback intervention, but did not independently revalidate the ANN or assess external generalizability; laboratory KAM outcomes were measured with motion capture and force plates. | Significant group-by-time interactions were observed for KAM1 (p<0.001), KOOS5 (p=0.033), and VASP (p=0.026), but not KAM2. Post-training between-group differences favored gait retraining for KAM1 (-0.39 %BW×height; p=0.005), VASP (-1.43/10; p=0.014), and KOOS5 (+8.72/100; p=0.007). Within the retraining group, KAM1 fell 16.5% and VASP 35.5% (both p<0.001). KAAI and KFM showed no significant group-by-time interaction. |
| **Panfilov et al., 2025**  *End-to-End Prediction of Knee Osteoarthritis Progression With Multimodal Transformers* | Five horizon-specific OAI datasets: 12 months, n=3,967 (227 progressors); 24 months, n=3,735 (315); 36 months, n=3,585 (425); 48 months, n=3,448 (501); and 96 months, n=2,421 (670). Site-D hold-out test sets contained 1,016, 933, 896, 867, and 626 participants, respectively. One knee per participant was selected. | Osteoarthritis Initiative (OAI), a multicenter longitudinal cohort. Baseline clinical data, weight-bearing radiographs, and multi-sequence 3-T knee MRI were linked to radiographic follow-up at 1, 2, 3, 4, and 8 years. | Retrospective AI model-development and evaluation study using prospectively collected longitudinal cohort data. Separate end-to-end models were developed for each progression horizon to compare clinical baselines, individual imaging modalities, and multimodal fusion configurations. | Radiographs were modeled with a ResNeXt-50 CNN. Each MRI sequence used a ResNet-50 slice encoder followed by a Transformer aggregator. Multimodal models used modality-specific CNN/Transformer branches and a final cross-modal Transformer; clinical variables entered through a shallow fully connected branch. Models were trained as five-fold ensembles using focal loss and minority-class oversampling. | Posteroanterior weight-bearing knee radiographs; sagittal 3D DESS structural MRI; coronal intermediate-weighted TSE structural MRI; sagittal T2 maps for compositional MRI; and clinical variables including age, sex, BMI, prior knee injury/surgery, WOMAC score, and baseline Kellgren-Lawrence grade. | Radiographic progression was worsening in Kellgren-Lawrence grade between baseline and the specified 12-, 24-, 36-, 48-, or 96-month visit. KLG 0 and 1 were pooled; knees with no grade change were controls, and knees showing radiographic improvement were excluded to avoid ambiguous trajectories. | All participants from OAI acquisition site D formed a fixed hold-out test set (approximately 25%-26% of each horizon dataset), partially testing acquisition-site domain shift. Remaining sites were used in stratified five-fold training/validation. Fold predictions were ensembled; ROC AUC and average precision were evaluated on the hold-out set with 1,000 stratified bootstrap iterations. No independent non-OAI external cohort was used. | The top-ranked imaging fusion model (radiograph+DESS+T2 map) achieved ROC AUCs of 0.76, 0.75, 0.70, 0.73, and 0.77 and average precision of 0.13, 0.16, 0.22, 0.27, and 0.57 at 12, 24, 36, 48, and 96 months, respectively. DESS alone achieved AUC 0.71-0.76 and AP 0.12-0.54. Multimodal fusion improved 1-year prediction but was not consistently superior to structural MRI across longer horizons. |
| **York et al., 2024**  *A Radiographic Artificial Intelligence Tool to Identify Candidates Suitable for Partial Knee Arthroplasty* | An initial archive of 6,000 radiographs yielded 1,241 technically adequate four-view unilateral knee series, comprising 4,967 labeled images used for analysis. Expert labels included 3,284 partial-knee-arthroplasty images, 792 no-arthroplasty images, 160 patellofemoral-arthroplasty images, and 731 total-knee-arthroplasty images. | Imperial College Healthcare Trust, London, UK. Pre-existing pseudonymized radiographs were obtained for adults imaged for suspected or confirmed knee OA without prior arthroplasty. | Retrospective single-center diagnostic/decision-support model-development study. Three high-volume partial-knee-arthroplasty surgeons independently reviewed each radiographic series and resolved disagreements by consensus to create the reference treatment label. | Six convolutional neural-network backbones were evaluated: Inception v3, ResNeXt-TS, EfficientNet-ES, EfficientNet-B2, MobileNet-v2, and DenseNet-121-xray. An exchangeable multi-view module averaged predictions from the four radiographic views. Training used cross-entropy with label smoothing, class weighting, AdamW optimization, early stopping, and ImageNet or chest-radiograph pretraining where applicable. | Four routine knee radiographic views: weight-bearing anteroposterior, Rosenberg, weight-bearing lateral, and skyline. DICOM images were converted to 512×512-pixel JPEGs and processed as unilateral knees. | The reference outcome was the expert-panel arthroplasty recommendation: no arthroplasty, medial or lateral partial knee arthroplasty, patellofemoral joint arthroplasty, or total knee arthroplasty. Reported performance tables combined medial and lateral partial arthroplasty into a PKA class. | Radiographic series were randomly divided by unique identifier into training, validation, and test subsets with probabilities of 70%, 15%, and 15%. Validation loss guided early stopping, and AUROC, F1 score, and class-specific accuracy were measured on the internal test subset. Initial independent expert agreement was 93.2%, but there was no external-site or prospective clinical validation. | EfficientNet-ES performed best for PKA identification: AUROC 0.9515, F1 0.8335, and accuracy 0.794. For no arthroplasty, its AUROC/F1/accuracy were 0.954/0.679/0.875; performance was lower for patellofemoral arthroplasty (0.785/0.3335/0.3335) and total knee arthroplasty (0.7365/0.3425/0.3275). Applicability to different populations, equipment, and radiographic protocols remains untested. |
| **van Helvoort et al., 2020**  *Cohort Profile: The Applied Public-Private Research Enabling OsteoArthritis Clinical Headway (IMI-APPROACH) Study* | 297 patients with predominantly tibiofemoral knee OA were included across five European centers: Utrecht, n=153; Leiden, n=50; A Coruña, n=43; Oslo, n=31; and Paris, n=20. The selection flow included 432 screened candidates drawn from existing cohorts and newly recruited outpatients. | Five European observational OA cohorts (CHECK, HOSTAS, MUST, PROCOAC, and DIGICOD), supplemented by newly recruited outpatients. The final APPROACH cohort was followed in the Netherlands, Spain, Norway, and France. | Prospective 2-year multicenter cohort profile with visits at baseline and 6, 12, and 24 months. Machine-learning enrichment was used to recruit participants with a high predicted probability of structural progression, pain progression, or both, followed by intensive multimodal phenotyping for future model validation and phenotype/endotype discovery. | Two-stage ML preselection was described. Cohort-specific models trained on longitudinal CHECK data first ranked candidates; a second uniform model reranked all screened candidates using harmonized screening variables. Separate structural and pain models were used. The cohort-profile paper did not report the algorithm family, hyperparameters, or a finalized post-follow-up predictive model. | Selection inputs included reduced KOOS pain/symptom/function measures, BMI, current and prior-week numeric pain ratings, age, sex, and radiographic Knee Image Digital Analysis parameters. Planned longitudinal inputs included radiographs, MRI, CT/whole-body low-dose CT, blood and urine biomarkers, gait/motion analysis, performance tests, clinical examination, and patient-reported outcomes. | Selection-model structural progression was JSW reduction ≥0.3 mm/year over 2-3 years. Pain progression/sustained pain met at least one criterion: KOOS pain worsening ≥5/year with score ≥40 at 2 years; worsening ≥10/year with score ≥35 at 2 years; or score ≥40 at both baseline and 2 years. Final cohort outcomes were planned to include observed structural, pain, functional, and phenotype/endotype changes over 2 years. | At publication, this was a baseline cohort-profile and selection-feasibility report. Prospective validation was planned by comparing preselection probabilities with observed 2-year progression; subsequent progression models were to be trained/tested using baseline and 6-month data and would require external validation. No completed prospective validation, calibration analysis, or independent external validation was reported. | No AUROC, accuracy, sensitivity, specificity, or calibration metric for the preselection model was reported in this cohort-profile article. The main reported result was operational feasibility of ML-enriched recruitment and assembly of a 297-patient high-risk cohort; actual utility of the prediction models and phenotype/endotype prediction awaited completion of the 2-year follow-up. |

**Sources of heterogeneity.** The evidence base is heterogeneous in cohort composition and disease severity; analytical unit (participant, knee, image, or scan); cohort source and recruitment strategy; imaging acquisition and MRI sequence; sensor configuration and laboratory versus real-world data collection; AI architecture and feature-engineering strategy; outcome definition (segmentation, diagnosis, radiographic progression, pain/function, treatment response, loading, or arthroplasty candidacy); prediction horizon; validation design (cross-validation, internal hold-out, site-based hold-out, or independent external cohort); and reported performance metric (e.g., Dice/IoU, AUROC/AUC, AP, F1, accuracy, correlation, MAE/RMSE, or clinical effect size). Direct numerical comparison across studies should therefore be interpreted cautiously.

**Abbreviations:** ADL, activities of daily living; AI, artificial intelligence; AIC, Akaike information criterion; AKS, American Knee Society score; ANN, artificial neural network; AP, average precision; ASSD, average symmetric surface distance; AUC, area under the receiver operating characteristic curve; AUC/AUROC, area under the receiver operating characteristic curve; BH, body height; BIC, Bayesian information criterion; BMF, Biomarker Factory; BMI, body mass index; BW, body weight; cGAN, conditional generative adversarial network; CHECK, Cohort Hip and Cohort Knee; CI, confidence interval; CNN, convolutional neural network; CT, computed tomography; CTX-II, urinary C-terminal cross-linked telopeptide of type II collagen; CTX-Iα, α-isomerized urinary C-terminal cross-linked telopeptide of type I collagen; DCS, Decision Conflict Scale; DESS, dual-echo steady-state; DNN, deep neural network; DSC, Dice similarity coefficient; EMG, electromyography; F1, harmonic mean of precision and recall; FE, finite element; FNIH, Foundation for the National Institutes of Health; FSE/TSE, fast/turbo spin echo; GADF, Gramian Angular Difference Field; GLM, generalized linear model; HADS, Hospital Anxiety and Depression Scale; HD95, 95th-percentile Hausdorff distance; HKA, hip-knee-ankle angle; ICC, intraclass correlation coefficient; ICF, International Classification of Functioning, Disability and Health; IMI-APPROACH, Innovative Medicines Initiative-Applied Public-Private Research enabling OsteoArthritis Clinical Headway; IMU, inertial measurement unit; IoU, intersection over union; JCF, joint contact force; JSL, joint-space loss; JSN, joint-space narrowing; JSW, joint-space width; K-DQI, Knee Osteoarthritis Decision Quality Instrument; KAAI, knee adduction angular impulse; KAM, knee adduction moment; KFM, knee flexion moment; KIDA, Knee Image Digital Analysis; KL/KLG, Kellgren-Lawrence grade; KOA, knee osteoarthritis; KOOS, Knee injury and Osteoarthritis Outcome Score; KOOS JR, Knee Injury and Osteoarthritis Outcome Score Joint Replacement; KR, knee replacement; LC-MS/MS, liquid chromatography-tandem mass spectrometry; LSTM, long short-term memory; MAE, mean absolute error; MCID, minimal clinically important difference; MJCF, medial joint contact force; ML, machine learning; MOST, Multicenter Osteoarthritis Study; MRI, magnetic resonance imaging; MRM, multiple reaction monitoring; NRS, numeric rating scale; OA, osteoarthritis; KOA/OA, knee osteoarthritis/osteoarthritis; OAI, Osteoarthritis Initiative; OR, odds ratio; PC/PCA, principal component/principal component analysis; PFJA, patellofemoral joint arthroplasty; PFOA, patellofemoral osteoarthritis; PKA, partial knee arthroplasty; PR-AUC, area under the precision-recall curve; PROMIS, Patient-Reported Outcomes Measurement Information System; PSI, patient-specific instrumentation; qMRI, quantitative magnetic resonance imaging; QOL, quality of life; RCT, randomized controlled trial; RMSE, root-mean-square error; RNN, recurrent neural network; ROC, receiver operating characteristic; SDM, shared decision-making; T2, transverse relaxation time; TFOA, tibiofemoral osteoarthritis; TKA, total knee arthroplasty; TRIPOD, Transparent Reporting of a Multivariable Prediction Model for Individual Prognosis or Diagnosis; TSE, turbo spin echo; UPLC-MS/MS, ultra-performance liquid chromatography-tandem mass spectrometry; VAE, variational autoencoder; VAS, visual analogue scale; VASP, visual analogue pain scale; VCA, valgus correction angle; WOMAC, Western Ontario and McMaster Universities Osteoarthritis Index; WORMS, Whole-Organ Magnetic Resonance Imaging Score; XR, radiography.
